# Supplementary material for: A simple model clarifies the complicated relationships of complex networks
Source: Sci Rep. 2014 Aug 27;4:6197. doi: 10.1038/srep06197 (PMC4145283; doi:10.1038/srep06197)
Supplement: Supplementary Information — SI [file srep06197-s1.pdf]

A simple model clarifies the complicated relationships of complex networks

Bojin Zheng[1,2,3,4], Hongrun Wu[3], Li Kuang[3],Jun Qin[1], Wenhua Du[1], Jianmin Wang[4], Deyi Li[4]

1. College of Computer Science, South-Central University For Nationalities, Wuhan 430074, China
2. State Key Laboratory of Networking and Switching Technology, Beijing University of Posts and Telecommunications, Beijing 100876, China
3. Computer School, Wuhan University, Wuhan 430072, China
4. School of Software, Tsinghua University, Beijing 100084, China

The supplementary material is divided into three parts: the theoretical analysis, the relationships and the experiments.

Part 1: Theoretical Analysis

This model is actually a multi-objective optimisation problem with constraints and a random variable. The model can be written as Equation (S1), which is the same as Equation (1) in the text.

$$\begin{cases} \min F_1(A) = \sum_{i=1}^{N} x_i(A) \\ \max F_2(A) = \sum_{i=1}^{N}\sum_{j=1}^{N} [x_i(A)]^a [x_j(A)]^b \delta_{ij}(A) \end{cases}$$
$$\textit{subject to}$$
$$(y-c)^2 = 0$$
$$N > x_i(A) \geq \text{xmin} \qquad \text{(S1)}$$

Here, $x(i)$ is the degree of node i of the complex network A, y is the average shortest path, and $c/xmin/a/b/N$ are non-negative constants. N is the number of nodes, and $xmin$ is the minimum degree. The function δ_{ij} indicates whether there is a link between node i and node j or not.

When considering multi-objective optimisation problems, the solutions are quite different from those of single-objective optimisation problems. We require the concept of 'the Pareto front' to discuss multi-objective optimisation.

For the convenience of discussion, we assume that all of the functions are to be minimised. For the maximised functions, we use a transform function to obtain the minimised function.

On the Pareto front

Multi-objective optimisation problems[1] differ from single-objective optimisation problems because the different objectives may conflict with each other. As Fig. S1 shows, the solution with the smallest value for the first objective exhibits one of the worst values for the second objective.

Therefore, the "best" solutions to a multi-objective optimisation problem can be defined as "none better is the best". All of the best solutions form a set, which is called the "non-dominated set" (NDS). None of the elements in the NDS are dominated by a feasible solution, and they form

the Pareto front. For convenience, the multi-objective optimal problems are commonly written as multiple maximum objectives or minimum objectives. Here, F_2 is larger than 1, so we rewrite equation S1 as S1'. In Fig.S1 and Fig.S5, the horizontal axis F_2 and vertical axis F1 is the form of S1'.

$$\begin{cases} \min F_1(A) = \sum_{i=1}^{N} x_i(A) \\ \min F_2(A) = \left(\sum_{i=1}^{N}\sum_{j=1}^{N}[x_i(A)]^a[x_j(A)]^b\delta_{ij}(A)\right)^{-1} \end{cases} \quad \text{(S1')}$$

subject to

$$(y-c)^2 = 0$$

$$N > x_i(A) \geq \text{xmin}$$

Taking Fig. S1 as an example, the red curve is the Pareto front, and the shadowed area is the feasible objective space. Every point in the shadowed area outside of the Pareto front is dominated by at least one point. Point C is dominated by point A because both F_1 and F_2 of point C are larger than F_1 and F_2 of point A. Even though F_1 of point B is the same as F_1 of point A, F_2 of point B is larger than F_2 of point A. Thus, Point B is also dominated by point A.

The Pareto front is called "the skyline" in the field of database management systems[2].

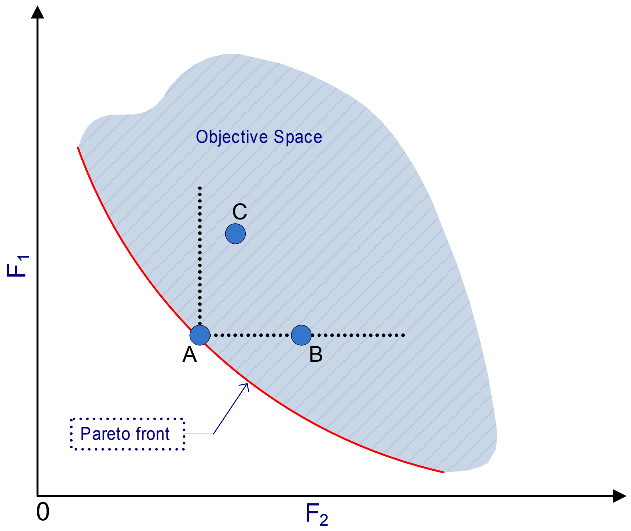

Fig. S1. A schematic map of the Pareto front

Mathematically, the dominance relationship and the Pareto front can be defined as follows.

Definition 1: [Dominance] For a given space Ω, A∈Ω, B∈Ω, $A(A_1,A_2,\ldots,A_n)$ is said to dominate $B(B_1,B_2,\ldots,B_n)$ (denoted as A p B) if and only if A is less than or equal to B for each

element but A≠B as shown in Equation (S2).

$$\forall i \in \{1,2,...,n\}, A_i \le B_i \wedge \exists j \in \{1,2,...,n\}, A_j < B_j \qquad \text{(S2)}$$

Definition 2: [The Pareto front] For a given space Ω, the Pareto front *PF* is defined as Equation (S3).

$$PF = \{u \mid \nexists v \in \Omega \wedge v \,\mathrm{p}\, u \wedge u \in \Omega\} \qquad \text{(S3)}$$

Detailed Analysis

Equation (S1) can be rewritten as Equation (S4).

$$\begin{cases} \min F_1(A) = \sum_{i=1}^{N} x_i \\ \max F_2(A) = \sum_{i=1}^{N}\sum_{j=1}^{N} x_i^a x_j^b \delta_{ij} \end{cases}$$
$$\textit{subject to} \qquad \text{(S4)}$$
$$(y-c)^2 = 0$$
$$N > x_i \ge \text{xmin}$$

Because *x(i)* and *x(j)* come from the same random variable X, assuming that the independently and identically distributed (iid) condition is satisfied, we use *x(i)* to approximate *x(j)*, so Equation (S4) can be rewritten as Equation (S5).

$$\begin{cases} \min F_1(A) = \sum_{i=1}^{N} x_i \\ \max F_2(A) = \sum_{i=1}^{N} x_i^a x_i x_i^b \end{cases}$$
$$\textit{subject to} \qquad \text{(S5)}$$
$$y = c$$
$$N > x_i \ge \text{xmin}$$

According to the iid condition, Equation (S5) can be rewritten as Equation (S6).

$$\begin{cases} \min f_1(x_i) = x_i \\ \max f_2(x_i) = x_i^{1+a+b} \end{cases}$$
$$\textit{subject to} \qquad \text{(S6)}$$
$$(y-c)^2 = 0$$
$$N > x_i \ge \text{xmin}$$

According to the constraint that the network should be connected, we have *x(i)* ≥1. Therefore,

Equation (S6) can be rewritten as the minimum style, as shown in Equation (S7).

$$\begin{cases} \min f_1(x_i) = x_i \\ \min f_2(x_i) = x_i^{-(1+a+b)} \end{cases}$$
$$\textit{subject to} \qquad \text{(S7)}$$
$$(y-c)^2 = 0$$
$$N > x_i \geq \text{xmin}$$

Based on the Lagrangian relaxation method, Equation (S7) can be rewritten as Equation (S8).

$$\begin{cases} \min f_1(x_i) = x_i + \theta(y-c)^2 \\ \min f_2(x_i) = x_i^{-(1+a+b)} + \theta(y-c)^2 \end{cases}$$
$$\textit{subject to} \qquad \text{(S8)}$$
$$N > x_i \geq \text{xmin}$$

For Equation (S8), the Pareto front can be rewritten[3] as Equation (S9) for each *x(i)*.

$$f_2(x_i) = x_i^{-(1+a+b)} \qquad \text{(S9)}$$

Let

$$p(x_i) = Cf_2(x_i) = Cx_i^{-(1+a+b)} \qquad \text{(S10)}$$

Here, C satisfies Equation (S11).

$$C = \frac{1}{\sum_{X=1}^{N-1} (X)^{-(1+a+b)}} \qquad \text{(S11)}$$

Note that Equation (S12) and Equation (S13) always hold, *x(i)* can be regarded as a sample of the random variable X, and p is a function defined on the sample space; therefore, p is the probability density function.

$$Cx_i^{-(1+a+b)} > 0 \qquad \text{(S12)}$$

$$\sum p = 1 \qquad \text{(S13)}$$

The probability density function satisfies Equation (S14).

$$p(X)=\frac{1}{\sum_{X=1}^{N\text{-}1}(X)^{-(1+a+b)}}(X)^{-(1+a+b)} \qquad \text{(S14)}$$

This section proves that the solutions of this model are scale-free networks. We refer to these scale-free networks as the optimal scale-free networks.

Note that F_1 is dependent on *xmin* and γ based on Equation (S15).

$$p(X)=\frac{1}{\sum_{X=xmin}^{N\text{-}1}(X)^{-\gamma}}(X)^{-\gamma} \qquad \text{(S15)}$$

When X=N-1, $p(X)$ is very small, and we can truncate it to a reasonable value of *xmax* that has a larger probability of occurring. Thus, Equation (S15) can be rewritten as Equation (S16).

$$p(X)=\frac{1}{\sum_{X=xmin}^{xmax}(X)^{-\gamma}}(X)^{-\gamma} \qquad \text{(S16)}$$

On the non-optimal scale-free networks

From a community structure perspective, a hub tend to connect many of the other nodes belonging to the same community, but the hubs of different communities are not linked together. To obtain optimal solutions, hubs will link together to obtain the largest F_2, so the community-structure networks are non-optimal solutions of this model.

The other non-optimal scale-free networks can be regarded as transitional forms between optimal scale-free networks and community-structure scale-free networks.

On the revised model for community-structure scale-free networks

Based on the Lagrangian relaxation method, the model can be rewritten as Equation (S17).

$$\begin{cases} \min F_1(A)=\sum_{i=1}^{N} x_i+\theta(y-c)^2 \\ \max F_2(A)=\sum_{i=1}^{N}\sum_{i=1}^{N} x_i^a x_j^b \delta_{ij}+\theta(y-c)^2 \end{cases}$$

subject to (S17)

$N > x_i \geq \text{xmin}$

When considering a network with community structure, we should consider the other distances in addition to the topological distance. We assume that the members of each community are categorized by the remainders of the modular function. Suppose that there exist two communities, where the nodes of odd numbers belong to the first community, and the nodes of even numbers belong to the second community. We can then rewrite the average distance of the network as Equation (S18).

$$y' = \sum_{i\%u \neq j\%u} \eta\delta_{ij} + y \qquad \text{(S18)}$$

Here, η is the distance penalty factor, and u is the number of communities.

Correspondingly, we define the constant of the average distance as Equation (S19).

$$c' = \Delta + c \qquad \text{(S19)}$$

Here, c is still the topological distance, and Δ represents other distances.

Therefore, the model for a community-structure network can be rewritten as Equation (S20).

$$\begin{cases} \min F_1(A)=\sum_{i=1}^{N} x_i+\theta(y'-c')^2 \\ \max F_2(A)=\sum_{i=1}^{N}\sum_{i=1}^{N} x_i^a x_j^b \delta_{ij}+\theta(y'-c')^2 \end{cases}$$

subject to (S20)

$N > x_i \geq \text{xmin}$

Using a structure that is similar to that of S1, the model for a community-structure network can be rewritten as Equation (S20').

$$\begin{cases} \min F_1(A)=\sum_{i=1}^{N} x_i \\ \max F_2(A)=\sum_{i=1}^{N}\sum_{i=1}^{N} x_i^a x_j^b \delta_{ij} \end{cases} \tag{S20'}$$

subject to

$$y' = c'$$

$$N > x_i \geq \text{xmin}$$

According to Equation (S18), because the model is similar to S1, we can easily prove that it can generate optimal solutions for community-structure scale-free networks in the revised form. Besides, because Equation (S18) predefines the distances of the nodes which imply two communities, the resulting networks should only have two communities. Because of the specific value of c', the resulting networks should only have one link between two communities. As to the more complicated and complex situations, a matrix of distances and the value of c' should be set deliberately.

Part 2: The Relationships

First, we explain the curve of the maximum of the average shortest path with $\gamma=2$, and then we illustrate the relationships of complex networks when $\gamma=3$.

On the maximum of the average shortest path

Fig. S2 is the same as Fig. 3 in the text.

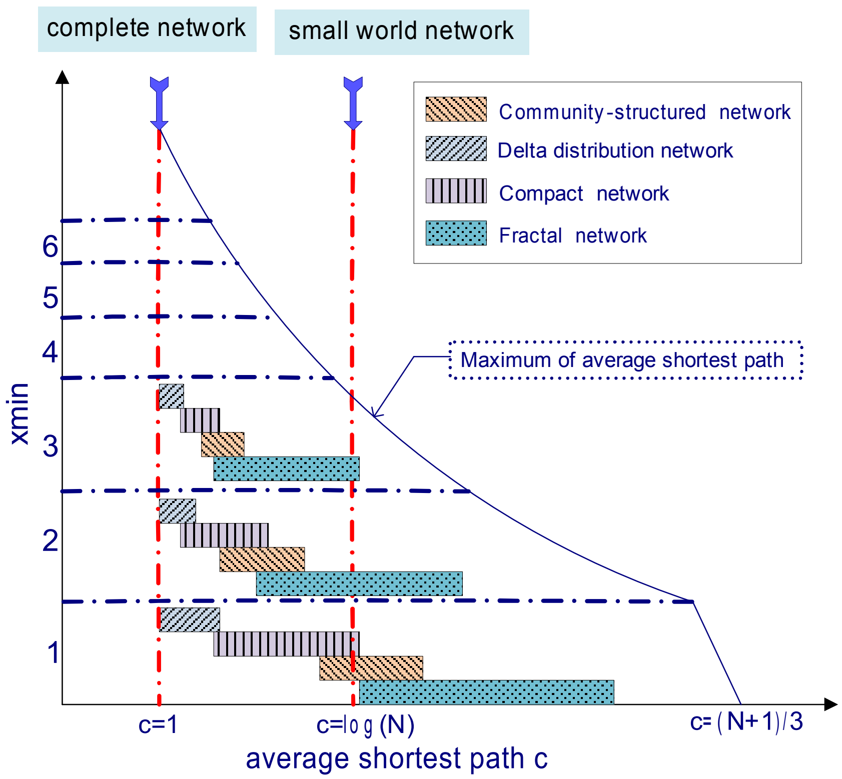

Fig. S2. A schematic map of the relationships among various complex networks when **γ**=2

When *xmin*=1, the maximum of the average shortest path (maxASP) is (N+1)/3 because the network must satisfy the constraint that the network be connected.

When *xmin*=2, the maximum of the average shortest path is $\frac{N^3-13N+24}{3N(N-1)}$, which is approximately (N+1)/3.

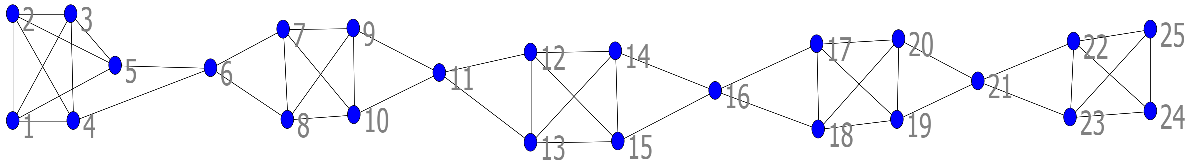

Fig. S3. A schematic network for a linear network when *xmin*=4

In S3 The network can be divided into some components with 5 nodes. Each node in the previous component have a distance of 3 hops to the corresponding node in the next component. For example, node 11 has 3 hops away from node 16. This rule also holds between node 12 and node 17, node 13 and node 18, node 14 and node 19, and node 15 and node 20.

Therefore, this network can be regarded as a linear network with *xmin*=1, but the weights of the edges are approximately 3. Thus, the maximum of the average shortest path satisfies Equation (S19).

$$maxASP \cong 3 \times \frac{\frac{N}{xmin+1}}{3} \cong \frac{N}{xmin+1} \quad \text{(S21)}$$

Moreover, Fig. S2 pre-assumes a smaller *N*. When *N* becomes larger, the schematic map may change slightly.

The relationships when γ =3

Here we draw the schematic map of the relationships of complex networks with the exponential value 3 as Fig. S4.

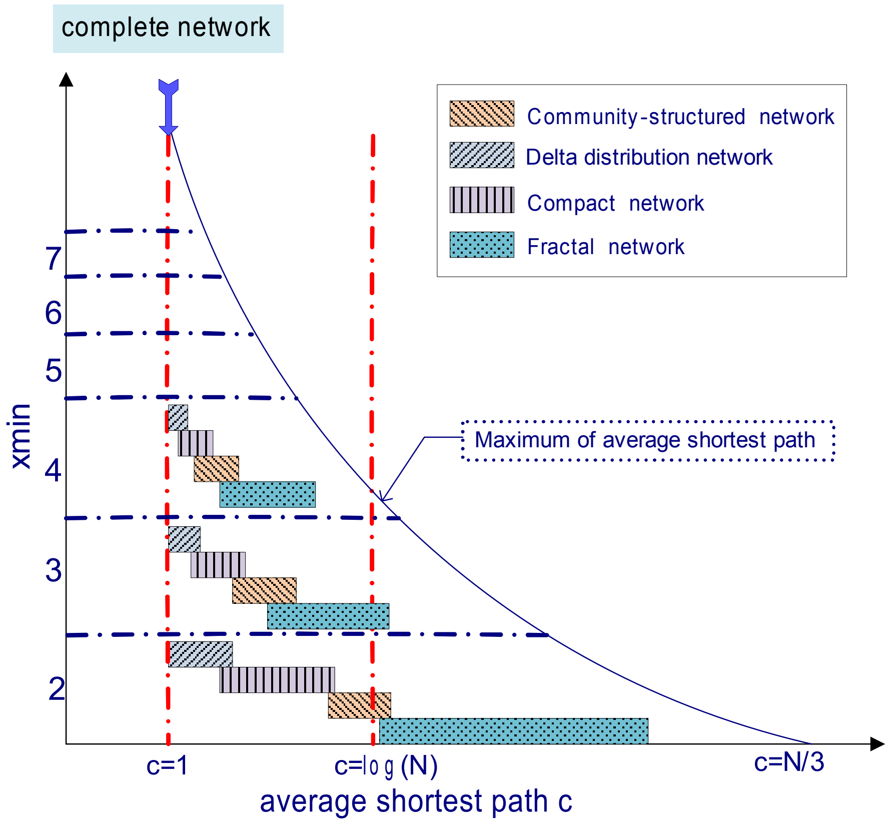

Fig. S4. A schematic map of the relationships of complex networks when γ=3

The relationships for γ = 3 are similar to those for γ = 2. However, xmin larger than 1. When γ = 3 and xmin=1, the network cannot be connected because there is a large number of nodes with only one neighbor.

Part 3: The Experiments

On the histogram method

In general, when dealing with a non-convex and discontinuous Pareto front, the histogram method does not guarantee that the obtained solutions are on the Pareto front. However, for the model in this paper, the histogram method is feasible.

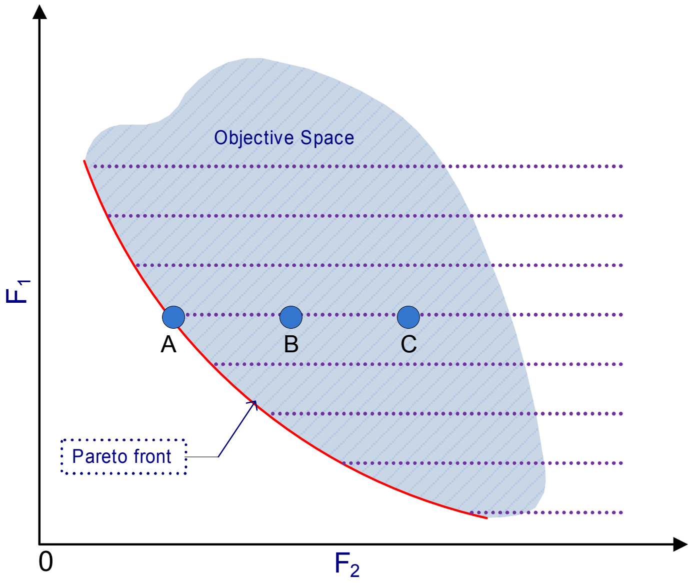

Fig. S5. A schematic map of the histogram method

As Fig. S5 shows, the histogram method fixes F_1 and then optimizes F_2. The feasible solutions move toward the Pareto front under the optimal objects, first C, then B and finally A. Because F_1 is discrete, this method is efficient. When F_1 decreases, F_2 increases; While $F1$ increase, $F2$ decrease. Therefore the solutions obtained by this method are on the Pareto front.

By using the histogram method for all of the different $F1$ values, we can obtain the entire Pareto front.

On the optimisation algorithm

This algorithm is designed to address a bi-objective optimisation problem with random variables.

To satisfy the iid condition, Equations (S15) and (S18) must be slightly modified. A weak constraint is added to ensure that the $x(i)$ are independent and identically distributed. This constraint should be very weak to ensure that the average shortest path can govern the optimisation process.

According to the results of the theoretical analysis, we use the standard power law distribution (*stP*) as a benchmark, and the degree distribution of the optimising network (*P*) must be approximated as close to stP as possible. Thus, Equation (S15) can be rewritten as Equation (S22).

$$\begin{cases} \min F_1^*(\mathrm{A})=\sum_{i=1}^{N} x_i+\theta(y-c)^2+\psi|stP-P| \\ \max F_2^*(\mathrm{A})=\sum_{i=1}^{N}\sum_{i=1}^{N} x_i^a x_j^b \delta_{ij}+\theta(y-c)^2+\psi|stP-P| \end{cases}$$

subject to (S22)

$N > x_i \geq \mathrm{xmin}$

Here, ψ is a constant that is much smaller than θ because the constraint of the average shortest path is much stronger than the constraint of distribution.

When using the histogram method, according to the iid, the bi-objective optimisation problem S22 can be transferred to a single objective optimisation problem as S23.

$$\min\ g(A)=\sum_{i=1}^{N} x_i^{-(a+b+1)}+\theta(y-c)^2+\psi\,|stP-P| \qquad \text{(S23)}$$

$N > x_i \geq \mathrm{xmin}$

Though benchmark is not a necessary condition, the optimisation process is much slow to converge if we do not use the benchmark to guide the algorithm.

✓ **Idea**

According to the equation of the power law distribution, which can be written as Equation (S16), we calculate the number of edges. Next, we attempt to delete one edge at random under the conditions of the *xmin* constraint and the connected network constraint; furthermore, we randomly choose two nodes without a link between them and then add a link between them. If g(A) becomes smaller, then we accept these changes. We repeat these attempts until a satisfactory result is obtained.

✓ **Pseudo-code**

1. Initialise the number of edges and the network A
2. Compute g(A) and let B=A
3. Do loop
4. Choose one edge at random from B
5. If the edge is valid to delete, then delete it
6. Otherwise, go to 3
7. Choose two nodes without a link between them from B; then, add a link
8. Compute g(B)

9. If g(B) < g(A), then let A=B
10. Until the terminal conditions are satisfied

✓ **Performance Analysis**

The algorithm is very simple. The time-consuming part is the calculation of the average shortest path. In our algorithm the time complexity of the average shortest path is *O(ElogV)* by Dijkstra algorithm with heap structure[4], where *V* is the number of nodes and *E* is the number of the edges..

The Experimental Results

We use 100 CPU kernels in personal computers to perform the experiments.

We set 24 parameter settings to illustrate the exact networks. For each parameter setting, the algorithm was performed 10 times. As the experimental results are very robust, we show only the results for the first run in Fig. S6- Fig. S29.

In all of the experiments, we set η =10 and θ =10. When obtaining the Delta-distribution networks, we set ψ =10^{-7}; when obtaining the random networks, we set ψ =0; in the other circumstances, we set ψ =10^{-5}.

For different parameter settings, the numbers of edges are different. According to Equation (S21), we list the numbers of edges that we have used in Table S1.

Table S1. The number of edges for different values of γ and *xmin*

γ	*xmin*	*E*
2	1	347
2	2	762
2	3	1157
3	1	Invalid
3	2	432
3	3	677
1	3	1200

For the experiments with γ =1, the number of edges is not in accord with Equation (S21) and is set with an estimate for a random network.

The figures show results for different values of γ. All the figures are combined by two parts.

The upper boxes demonstrate the degree distributions, and the fitting results *xmin*, *y*, γ are given. To reduce the noise, the fitting line in the upper boxes are plotted by log-bins method. Because the information of degree distributions will be lost by bining the data, so the fitting results(*xmin*, *y*, γ) are fitted from the original distributions.

When the degree distribution is power law, the degree distributions with blue circle fit well with the red line, and the fitting result *xmin* equals the parameter *xmin*.

The fractal networks checked by the box-covering method[6] are shown in Fig. S44-Fig. S47.

✓ Figures for γ =2

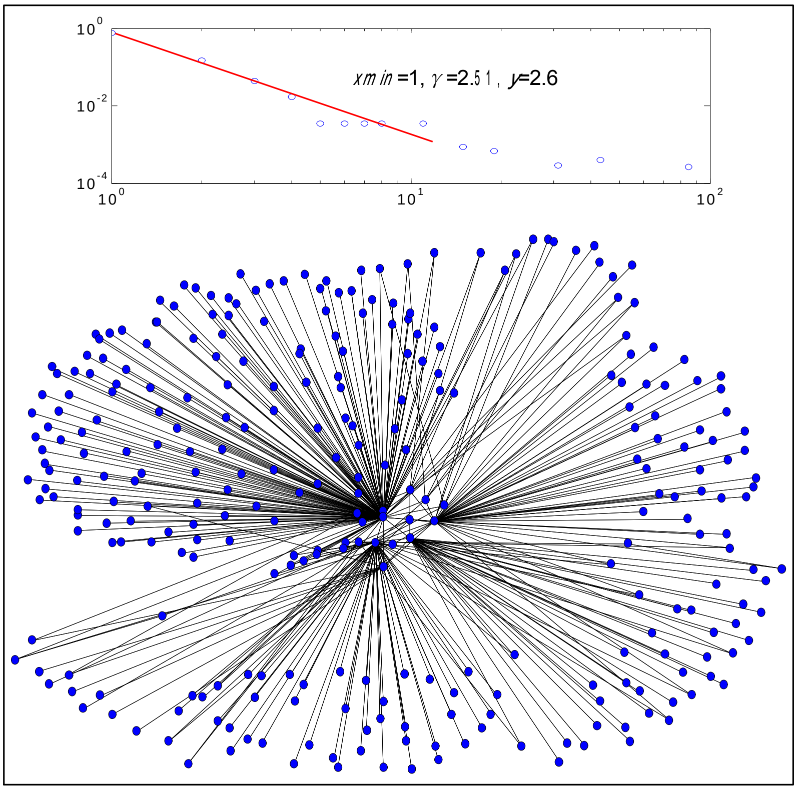

Fig. S6. A Delta-distribution network with *xmin*=1, *a*=0, *b*=1, and *c*=2.6

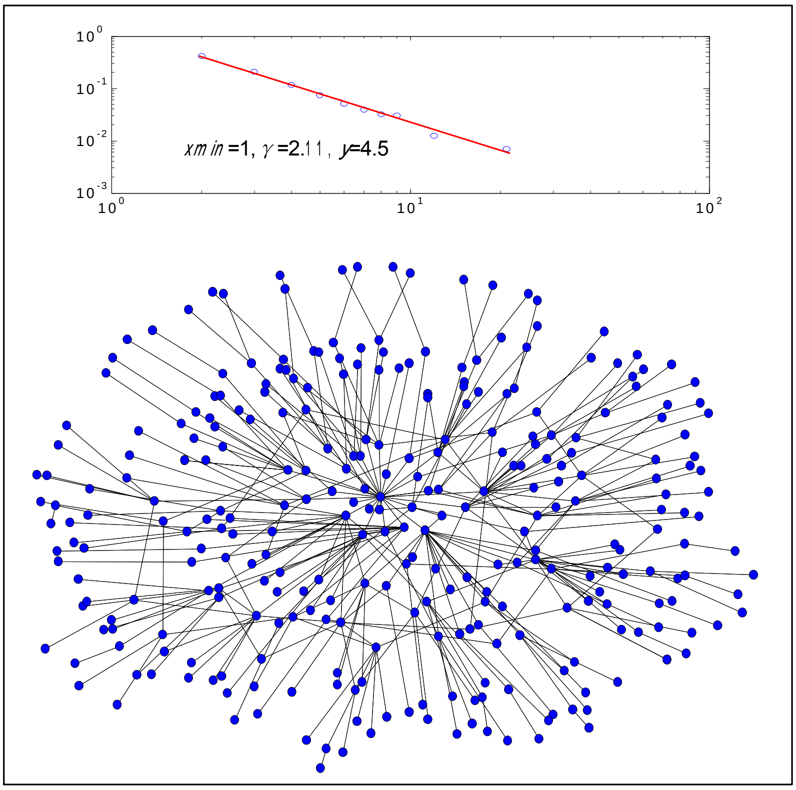

Fig. S7. A compact network with *xmin*=1, *a*=0, *b*=1, and *c*=4.5

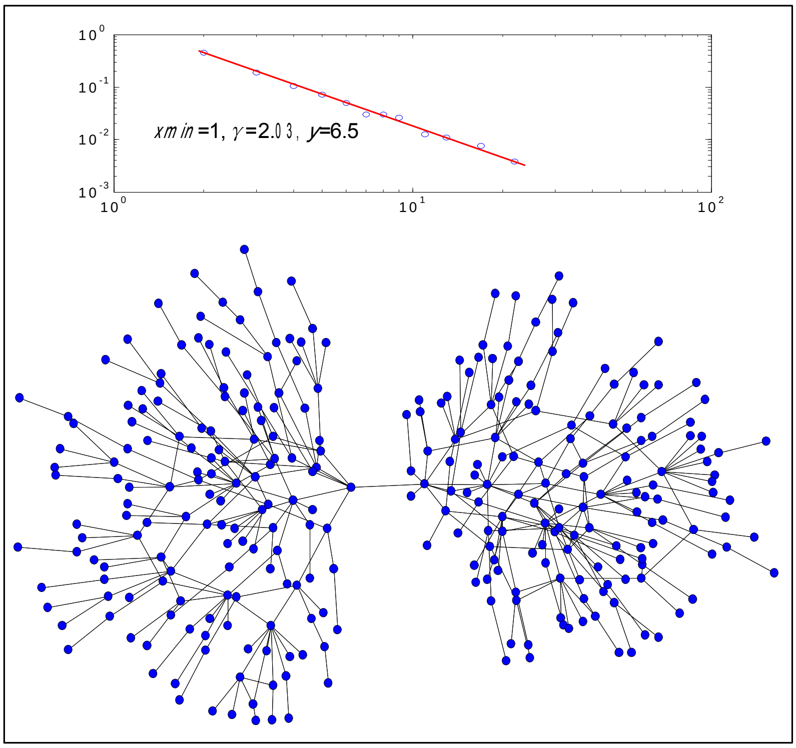

Fig. S8. A community-structure network with *xmin*=1, *a*=0, *b*=1, and *c*=6.5

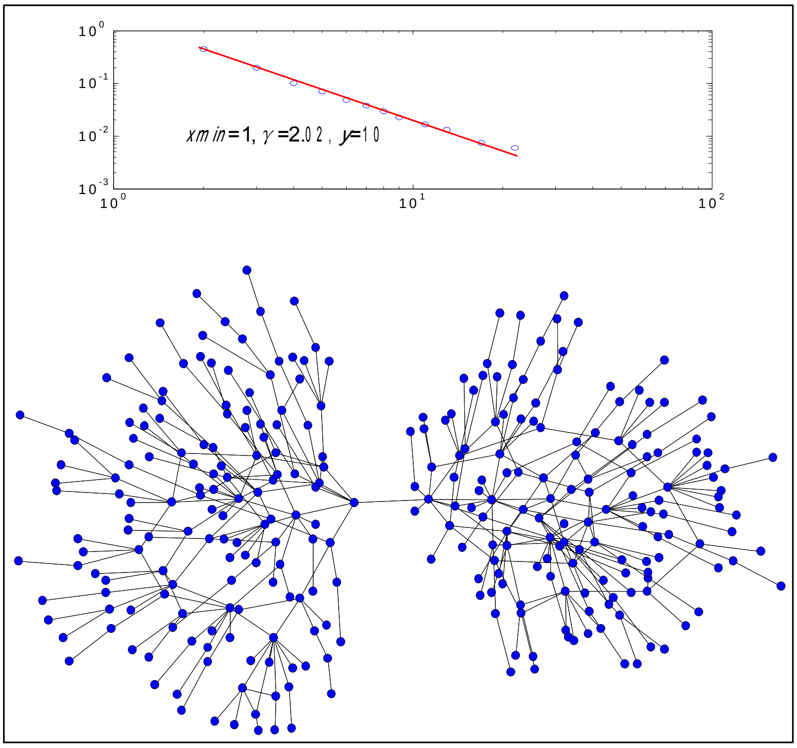

Fig. S9. A fractal network with *xmin*=1, a=0, *b*=1, and *c*=10

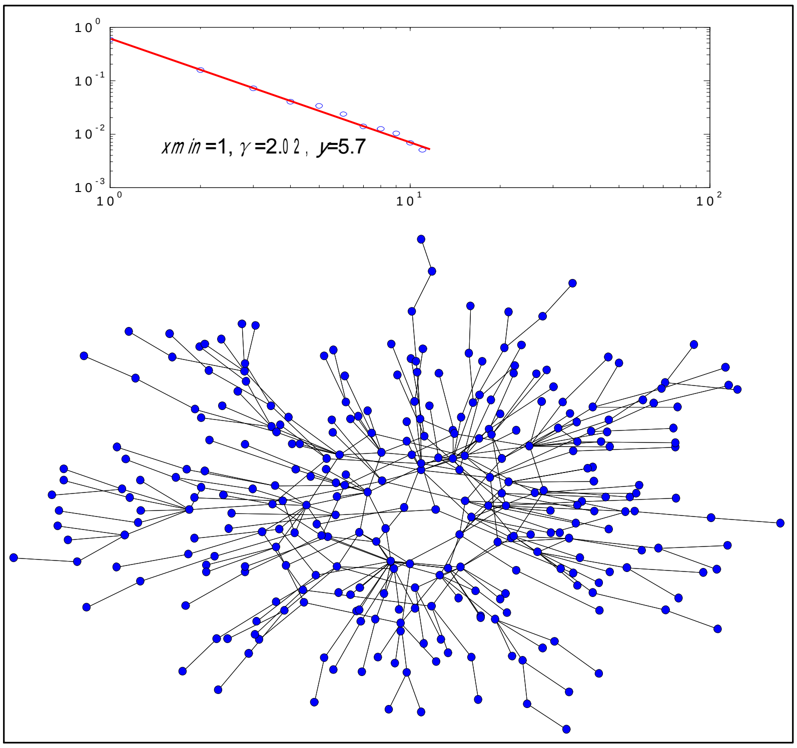

Fig. S10. A small-world network with *xmin*=1, *a*=0, *b*=1, and *c*=log(N)

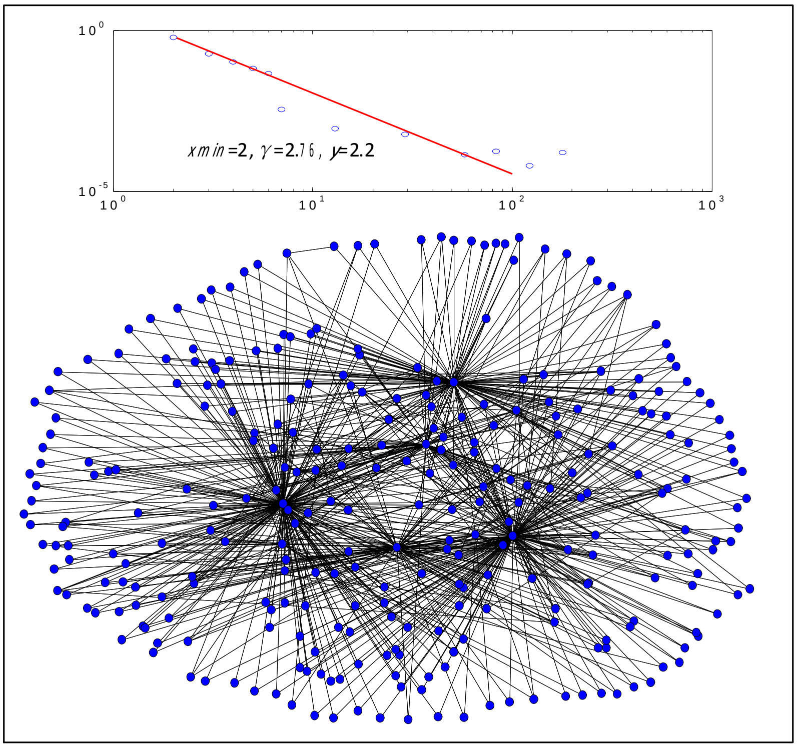

Fig. S11. A Delta-distribution network with *xmin*=2, *a*=0, *b*=1, and *c*=2.2

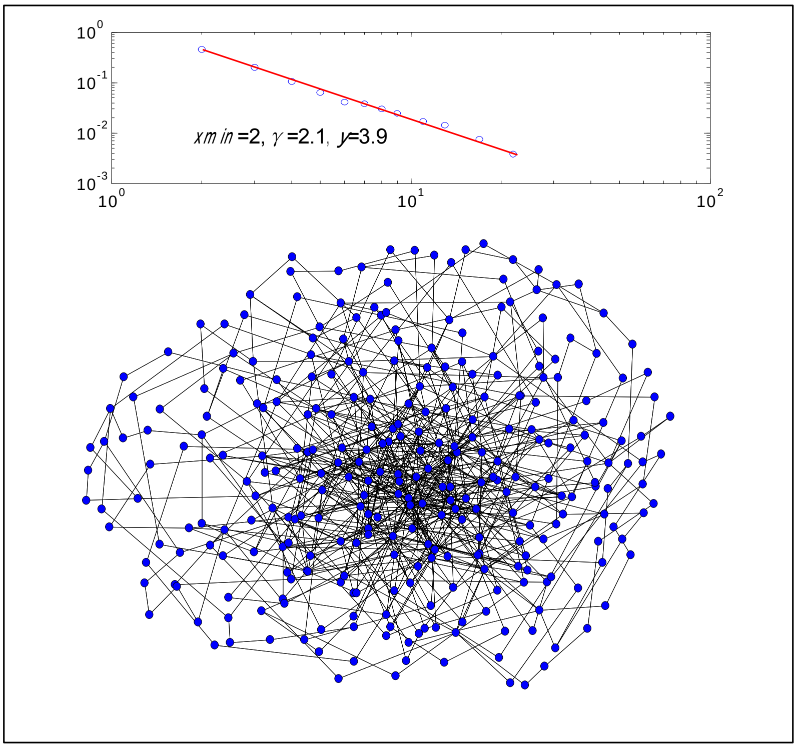

Fig. S12. A compact network with *xmin*=2, *a*=0, *b*=1, and *c*=3.9

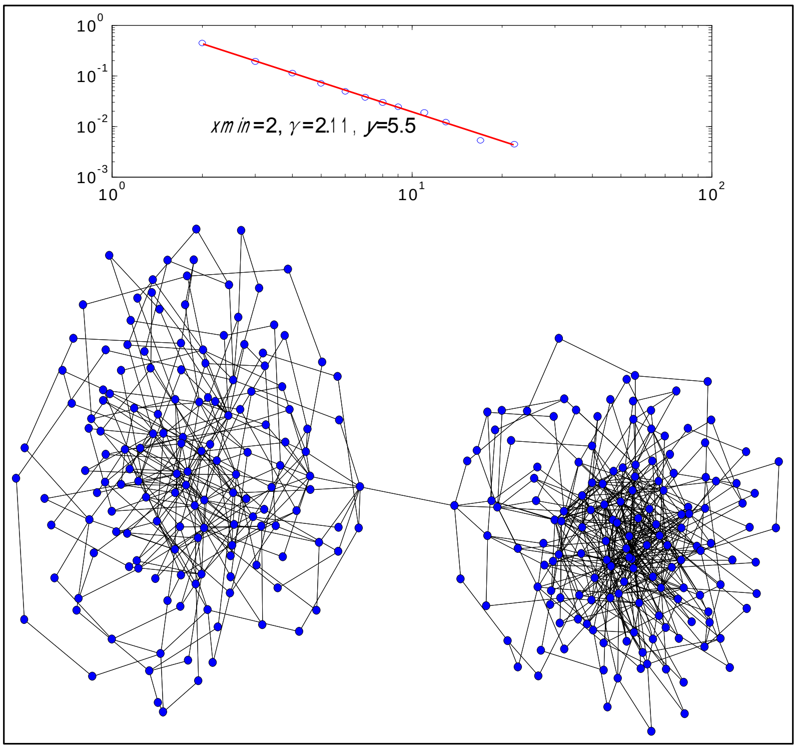

Fig. S13. A community-structure network with *xmin*=2, *a*=0, *b*=1, and *c*=5.5

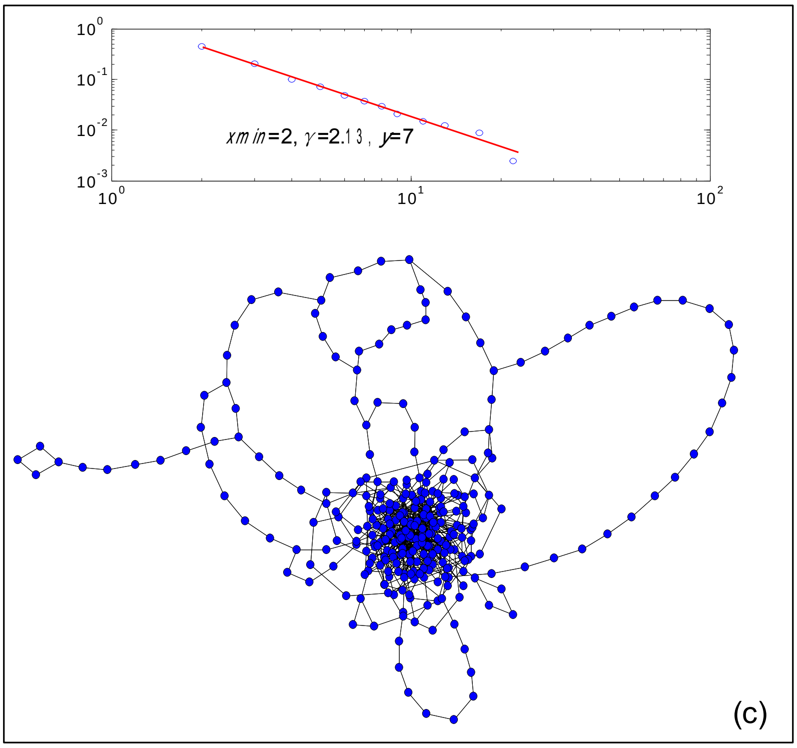

Fig. S14. A fractal network with *xmin*=2, *a*=0, *b*=1, and *c*=7

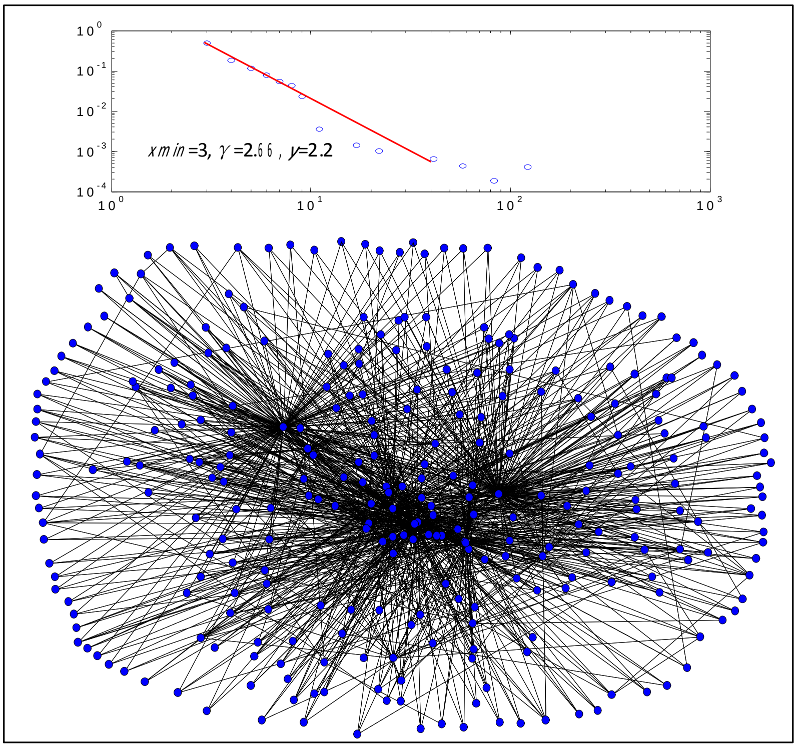

Fig. S15. A Delta-distribution network with *xmin*=3, *a*=0, *b*=1, and *c*=2.2

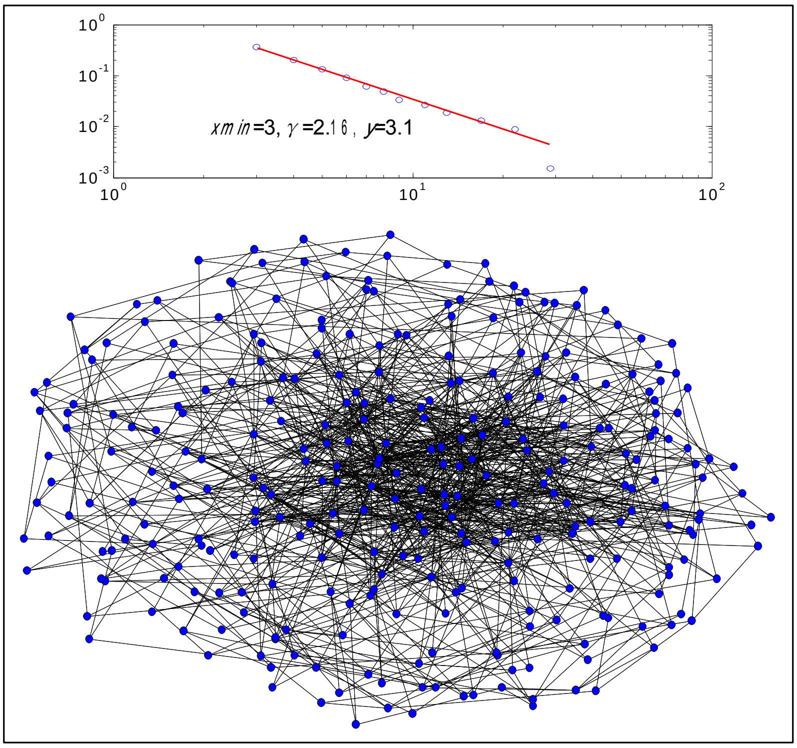

Fig. S16. A compact network with *xmin*=3, *a*=0, *b*=1, and *c*=3.1

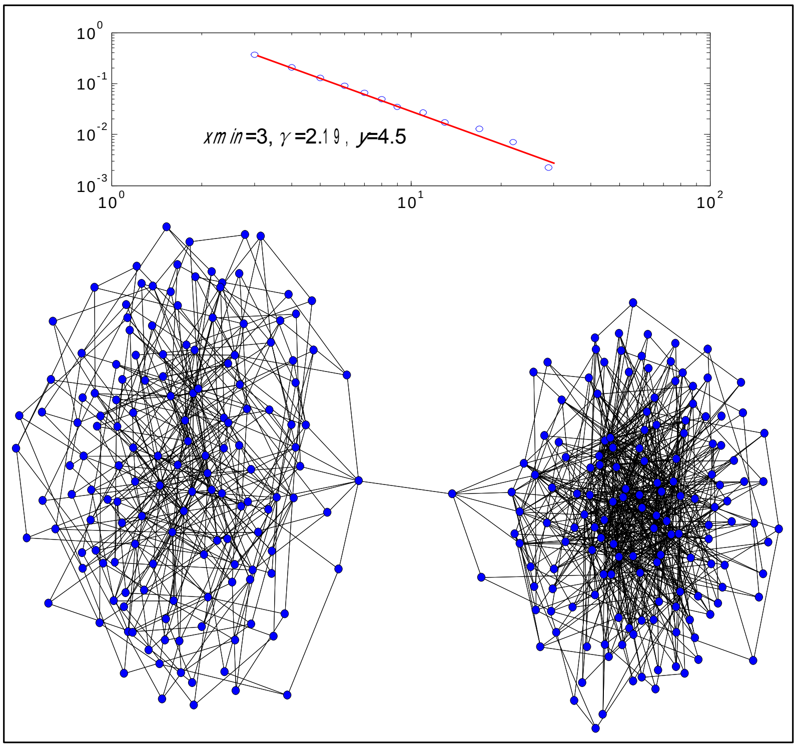

Fig. S17. A community-structure network with *xmin*=3, *a*=0, *b*=1, and *c*=4.5

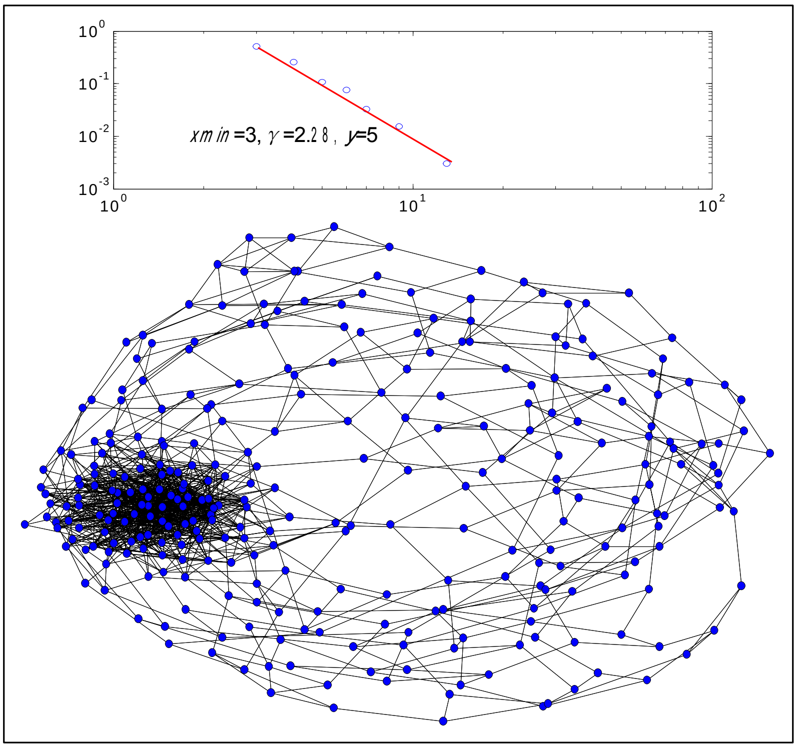

Fig. S18. A fractal network with *xmin*=3, *a*=0, *b*=1, and *c*=5

✓ Figures for γ =3

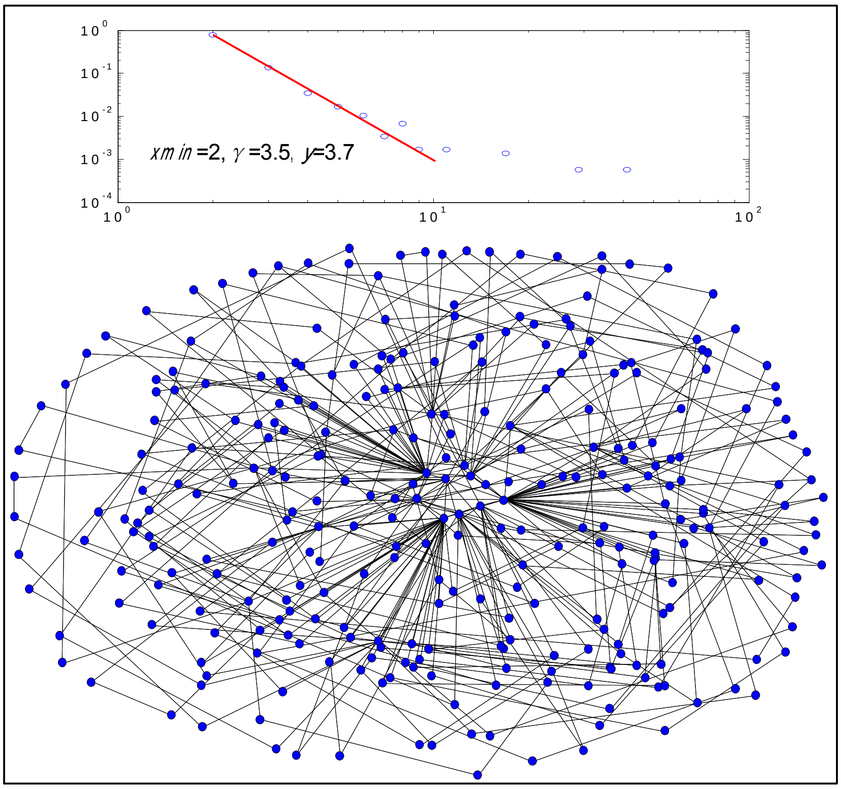

Fig. S19. A Delta-distribution network with *xmin*=2, *a*=1, *b*=1, and *c*=3.7

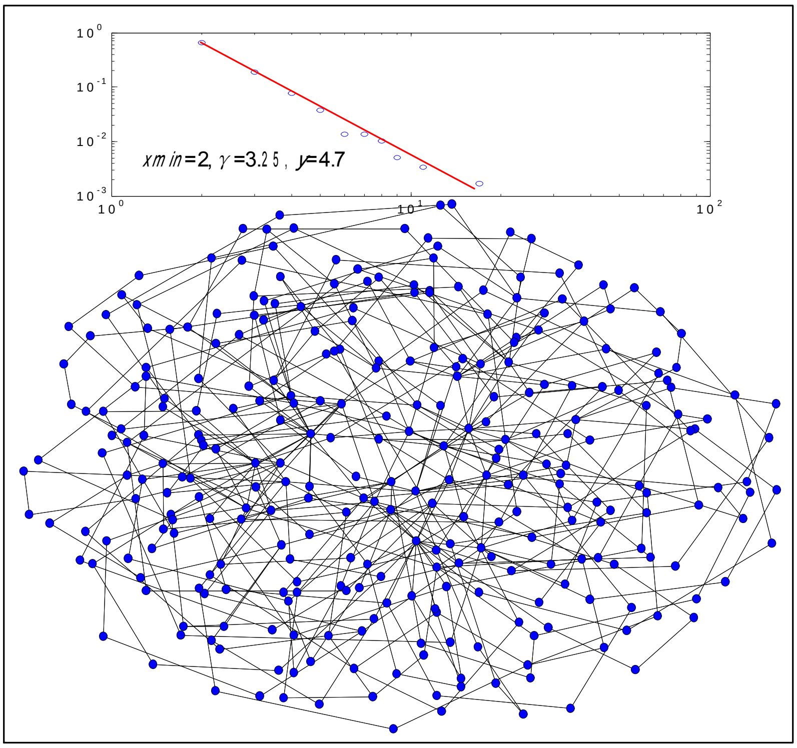

Fig. S20. A compact network with *xmin*=2, *a*=1, *b*=1, and *c*=4.7

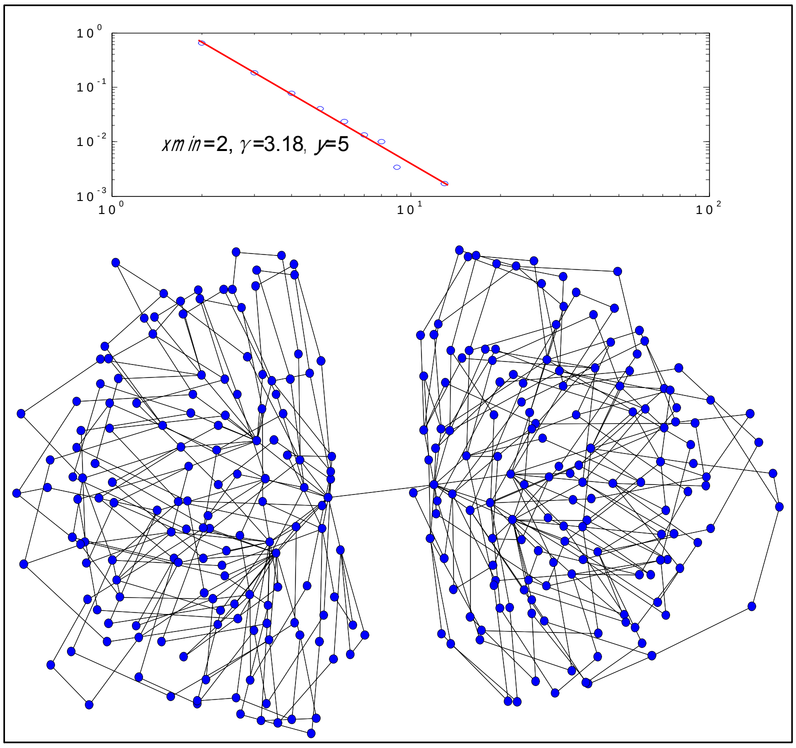

Fig. S21. A community-structure network with *xmin*=2, *a*=1, *b*=1, and *c*=5

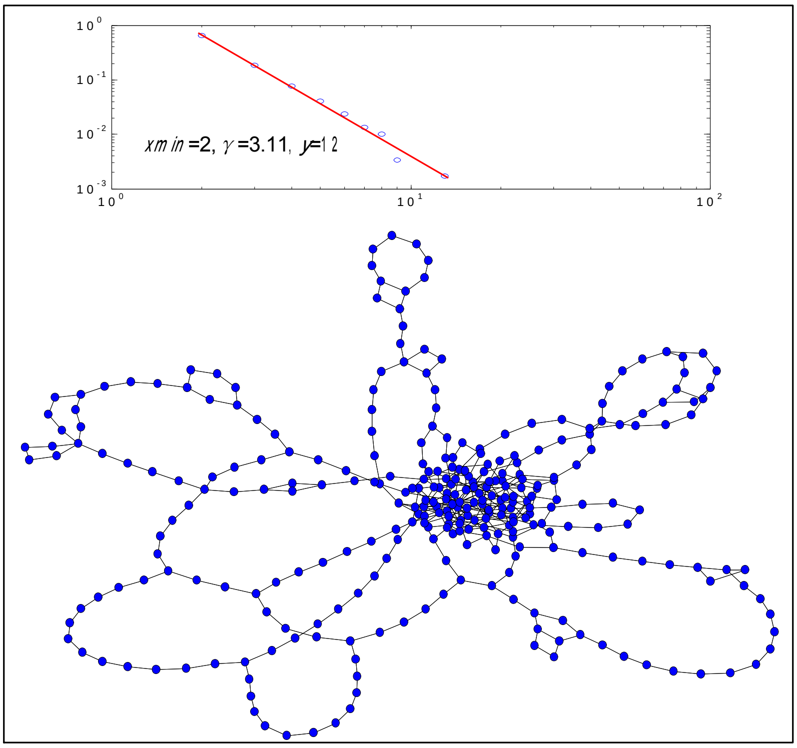

Fig. S22. A fractal network with *xmin*=2, *a*=1, *b*=1, and *c*=12

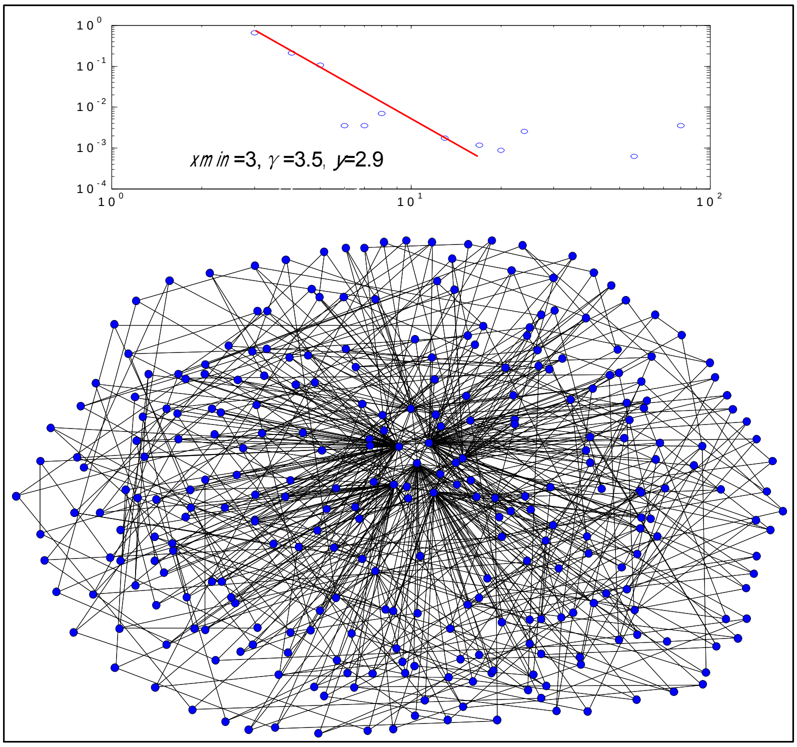

Fig. S23. A Delta-distribution network with *xmin*=3, *a*=1, *b*=1, and *c*=2.9

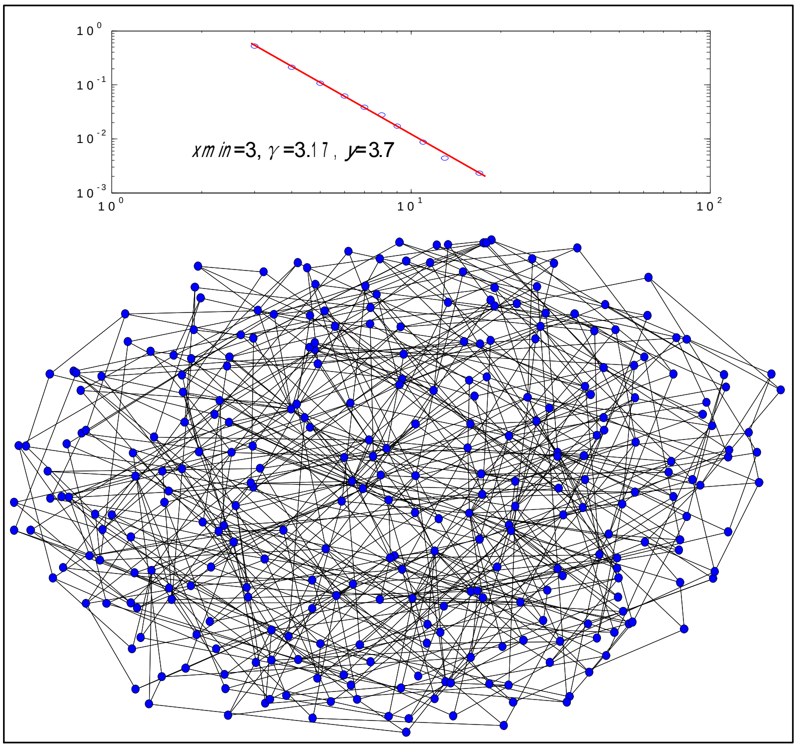

Fig. S24. A compact network with *xmin*=3, *a*=1, *b*=1, and *c*=3.7

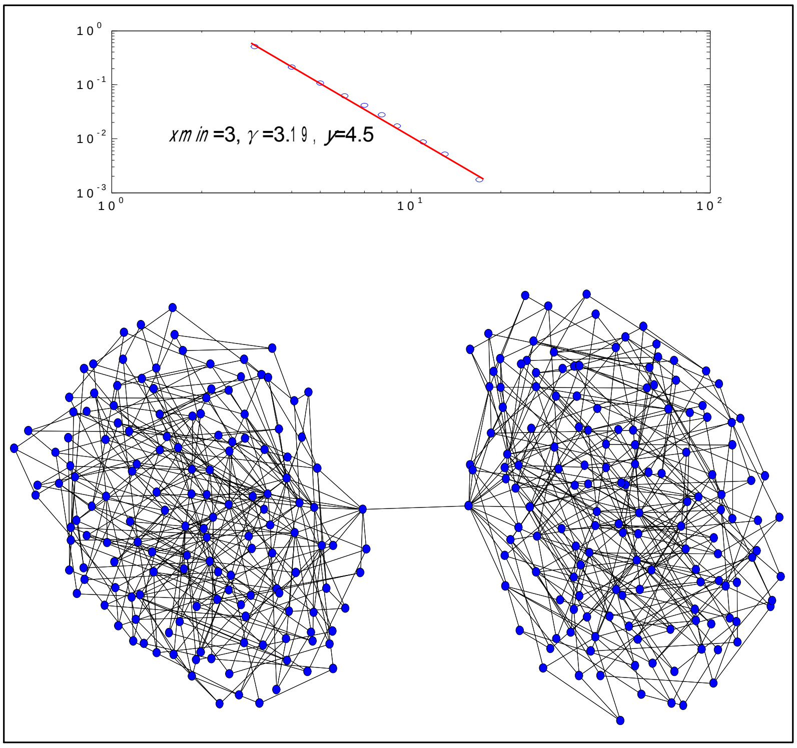

Fig. S25. A community-structure network with *xmin*=3, *a*=1, *b*=1, and *c*=4.5

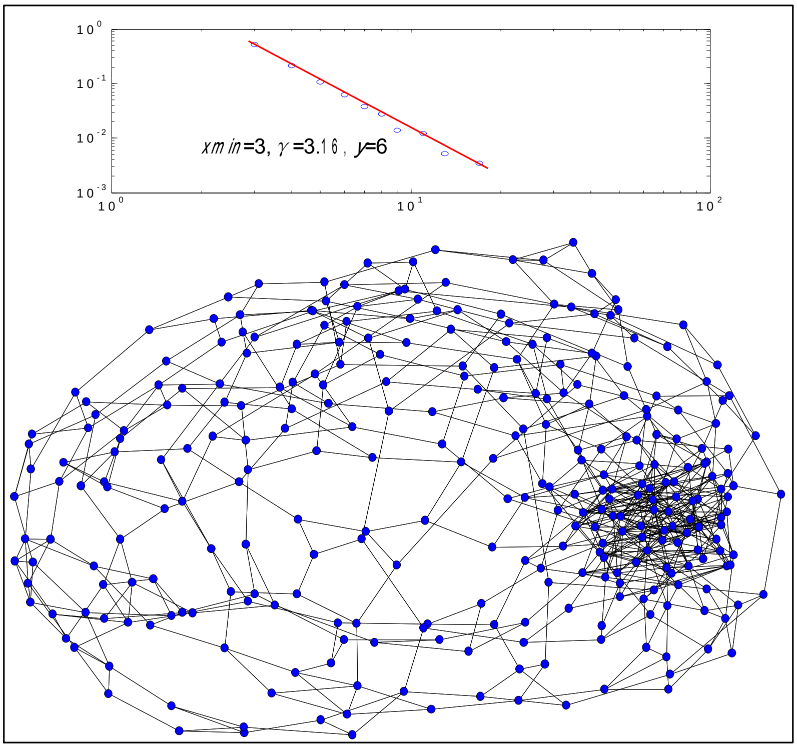

Fig.S26. A fractal network with *xmin*=3, *a*=1, *b*=1, and *c*=6

✓ Figures for $\gamma=1$

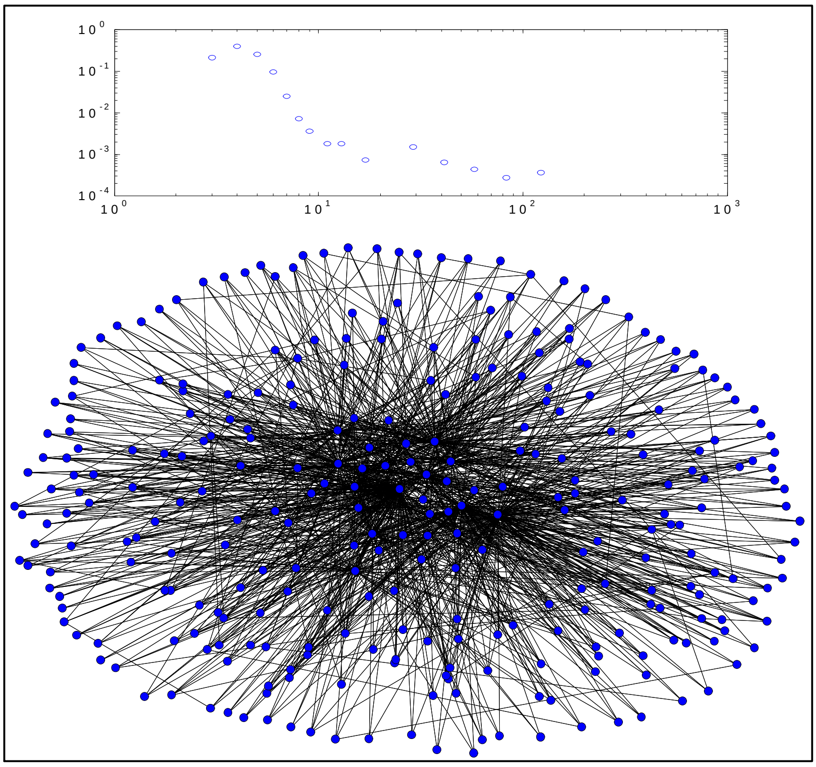

Fig. S27. A Delta-distribution and random network with *xmin*=3, *a*=0, *b*=0, and *c*=2.2

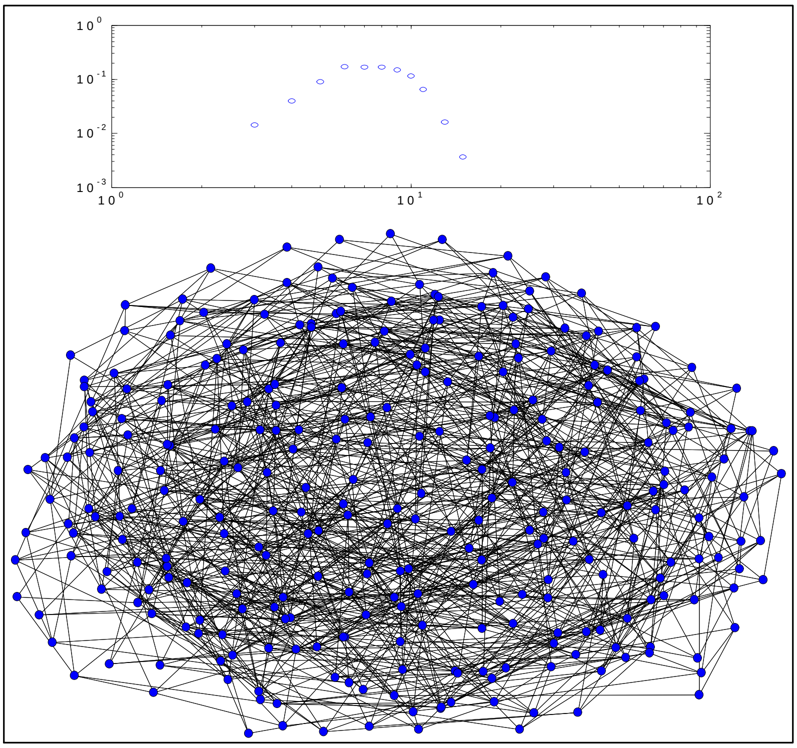

Fig. S28. A standard random network with *xmin*=3, *a*=0, *b*=0, and *c*=3.

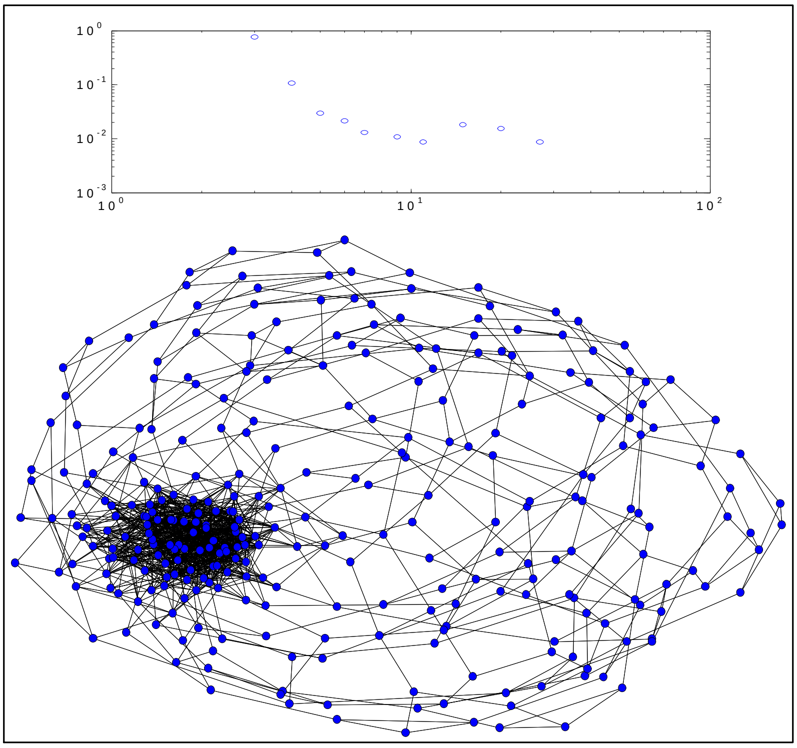

Fig. S29. A stretched random network with *xmin*=3, *a*=0, *b*=0, and *c*=5

For all 24 of the demonstrated networks, we calculated their fitness to the power law distribution and the other distributions[7]. According to the experimental data, we also listed the expected distributions and corresponding quantitative estimation to the power law distribution as shown in Table S2. Moreover, group 1 was related to Fig. S6, group 2 was related to Fig. S7 and so on.

In Table S2, the value of the third column "power-law" is the testing value of power law's plausibility, and the power law is considered a plausible statistical hypothesis for the distribution when $p \geq 0.1$, or the distribution is considered as non-power-law distribution. The values "*LR*" and "*p*" in 4-7 columns are used to compare the power law to alternative heavy-tailed distributions("exp.", "the stretched exp.", "the stretched exp." and "the stretched exp.") via a likelihood ratio test. "*LR*" is the log-likelihood ratio versus the alternative, if the LR is positive then the distribution is power law, if the *LR* is negative then the data is favored to be alternative distributions. Moreover, the significance of *LR* depends on the responding value *p*, when p<0.1, the *LR* is significant, or *LR* is not reliable, i.e., *LR* cannot be used to test whether power-law distribution is favored over the other one. The column "exp." represents the exponential distribution; "the stretched exp." represents the stretched exponential distribution; "log-normal"

represents the log-normal distribution; "power law + cutoff" represents the power law distribution with a cutoff; "status" represents the quantitative results supporting the power law distribution, and "expected distribution" represents the optimised degree distribution.

With regard to the statuses, "none" means that the degree distribution of the network was not in accordance with the power-law; "weak" means that the degree distribution fit the power law distribution well, but the alternatives are better; "moderate" means that the degree distribution fit the power law distribution very well, but the alternatives are also plausible; and "good" means that the degree distribution fit the power law distribution very well, and none of the alternatives are plausible. For example, the power-law value of group Fig.S16(the first row) is a plausible fit because the p value larger than 0.1, but the stretched exponential, log-normal and power-law + distributions are still plausible.

Table S2. Indices of the demonstrated networks

Group	index	power-law	exp.	stretched exp.	log-normal	power law + cutoff	status	expected distribution
Fig.S6	LR	-	12.418	-0.012	-0.747	-0.101	moderate	Delta
	p	0.500	0.000	0.913	0.387	0.919		
Fig.S7	LR	-	19.254	-2.384	-6.696	-2.155	weak	power-law
	p	0.505	0.000	0.123	0.010	0.031		
Fig.S8	LR	-	0.485	-0.278	0.205	-0.476	moderate	power-law
	p	0.116	0.486	0.598	0.651	0.634		
Fig.S9	LR	-	-0.678	1.73	3.793	0.666	moderate	power-law
	p	0.252	0.41	0.188	0.051	0.506		
Fig.S10	LR	-	4.914	-7.683	-10.852	-3.107	weak	power-law
	p	0.118	0.027	0.006	0.001	0.002		
Fig.S11	LR	-	30.87	-14.142	-13.572	4.981	none	Delta
	p	0.000	0.000	0.000	0.000	0.000		
Fig.S12	LR	-	-0.713	0.351	-0.501	1.859	moderate	power-law
	p	0.225	0.398	0.553	0.479	0.063		
Fig.S13	LR	-	-1.283	0.601	-0.255	1.436	moderate	power-law
	p	0.183	0.257	0.438	0.614	0.151		
Fig.S14	LR	-	14.483	24.429	21.273	0.986	moderate	power-law
	p	0.126	0.000	0.000	0.000	0.324		
Fig.S15	LR	-	-4.039	-19.421	-15.139	2.271	none	Delta
	p	0.000	0.044	0.000	0.000	0.023		
Fig.S16	LR	-	26.677	21.822	10.062	5.507	good	power-law

	p	0.102	0.000	0.000	0.002	0.000		
Fig.S17	LR	-	-0.091	0.42	0.093	2.081	moderate	power-law
	p	0.428	0.762	0.517	0.761	0.037		
Fig.S18	LR	-	-0.421	-0.25	-0.453	1.116	moderate	power-law
	p	0.263	0.516	0.617	0.501	0.265		
Fig.S19	LR	-	79.950	89.548	58.876	8.194	none	Delta
	p	0.000	0.000	0.000	0.000	0.000		
Fig.S20	LR	-	58.335	-3.563	-3.54	7.757	weak	power-law
	p	0.114	0.000	0.000	0.000	0.000		
Fig.S21	LR	-	47.212	51.619	30.309	5.931	good	power-law
	p	0.282	0.000	0.000	0.000	0.000		
Fig.S22	LR	-	42.975	48.962	22.105	5.500	good	power-law
	p	0.121	0.000	0.000	0.000	0.000		
Fig.S23	LR	-	5.615	19.237	20.873	2.629	none	Delta
	p	0.059	0.018	0.000	0.000	0.009		
Fig.S24	LR	-	1.529	3.581	0.628	2.687	moderate	power-law
	p	0.849	0.216	0.058	0.428	0.007		
Fig.S25	LR	-	-0.234	8.741	2.351	1.143	moderate	power-law
	p	0.277	0.629	0.003	0.125	0.253		
Fig.S26	LR	-	1.547	3.617	0.628	2.660	moderate	power-law
	p	0.843	0.214	0.057	0.428	2.66		
Fig.S27	LR	-	83.321	91.87	45.241	8.492	none	Delta
	p	0.000	0.000	0.000	0.000	0.000		
Fig.S28	LR	-	-25.36	-99.354	-94.68	1.517	none	random
	p	0.000	0.000	0.000	0.000	0.129		
Fig.S29	LR	-	47.394	34.029	35.936	1.637	none	random
	p	0.000	0.000	0.000	0.000	0.102		

According to Table S2, for lower values of N, all of the networks that were expected to present the power-law distribution were given a satisfactory quantitative estimation. In group 1, the network was estimated as the power-law distribution, which is the delta distribution that is very similar to the power law distribution.

On the Fast Algorithm

The hill-climbing algorithm wastes time on the validation of possible solutions, for example, to assure that the solution networks are connected and the degree of every node is greater than *xmin*. Moreover, the edge-exchanged strategy focuses on the edges; thus, the size of the search space is approximately N^2. Thus, a significant amount of time would be consumed under this

strategy.

If the degree distribution is known, a new algorithm will be more efficient. This proposed new algorithm first initialises a network with a known degree distribution, assures that the network has a large average shortest path length, and then exchanges the nodes to optimise the objectives, until a satisfactory solution is obtained.

Here, we depict the pseudocode and introduce the network initialisation method and node exchange method.

✓ **Pseudo-code**

1. Initialise the network A
2. Compute g(A) and let B=A
3. Do loop
4. Choose two nodes at random from B
5. Exchange the edges of the selected two nodes randomly
6. If g(B) < g(A), then let A=B
7. Repeat until the terminal conditions are satisfied

✓ **Network Initialisation Method**

The fast algorithm needs to initialise the solution with large average shortest path length and the demanded degree distribution.

Because linear networks have a large average shortest path length, the satisfactory initial networks can be the linear networks with a specific degree distribution. Here we use the scale-free linear networks as an example.

First we generated the samples based on the power law. Second, every node was given *xmin* neighbors, and they were chained together to form a linear network. Finally, additional links were added into the linear network, and make the degree distribution was allowed to follow the power law distribution.

Because the initial network was based on a linear network, its average shortest path length was quite large. Because F_2 was maximised, when the additional links were added, the hub nodes were allowed to link together.

✓ Node Exchange Method

The node exchange method randomly exchanges the links of two nodes. If node A and node B exchange their edges, the minimal possible exchange number, i.e., the smaller number of the degrees of two nodes, can be calculated. We set m edges to exchange. Here m is smaller than the minimal possible exchange number. For every exchange, we let the neighbour of node A, which was identified by a certain edge move to the neighbour of node B, and correspondingly, let the neighbour of node B move to the neighbour of node A, except in neighbourhood that existed previously. In the following simulations, m was set to 1.

According to the node exchange method, the degree distribution will never change in the process of optimisation. Therefore, we need to know the degree distribution in advance.

Moreover, if the initial network is connected , the node exchange method will never lead to the disconnection of the solution network. Therefore, the proposed method saves time by avoiding validation.

We used the proposed fast algorithm to obtain the solutions when the degree distribution was scale-free. We performed six groups of experiments to explore the network structure when N=1500. Every group was carried out 10 times to check the robustness of the algorithm. The experimental parameters are listed in Table S3.

Table S3. The parameters of the six groups of experiments

group	$Xmin$	γ	c
(a)	1	2	7.3
(b)	2	2	3.5
(c)	2	3	20
(d)	2	2	20
(e)	3	2	3.5
(f)	3	3	20

For each group, we chose the first solution network as an example, as shown in Fig. S30 - Fig. S35.

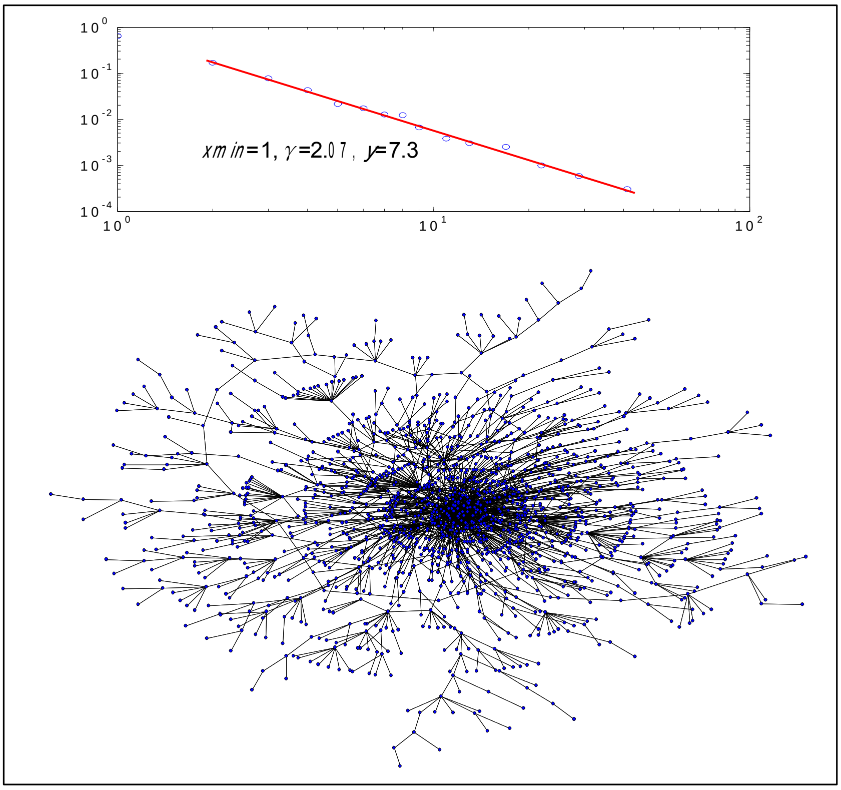

Fig. S30. Group (a). A small-world scale-free network with *xmin*=1, γ=2 and *c*=7.3

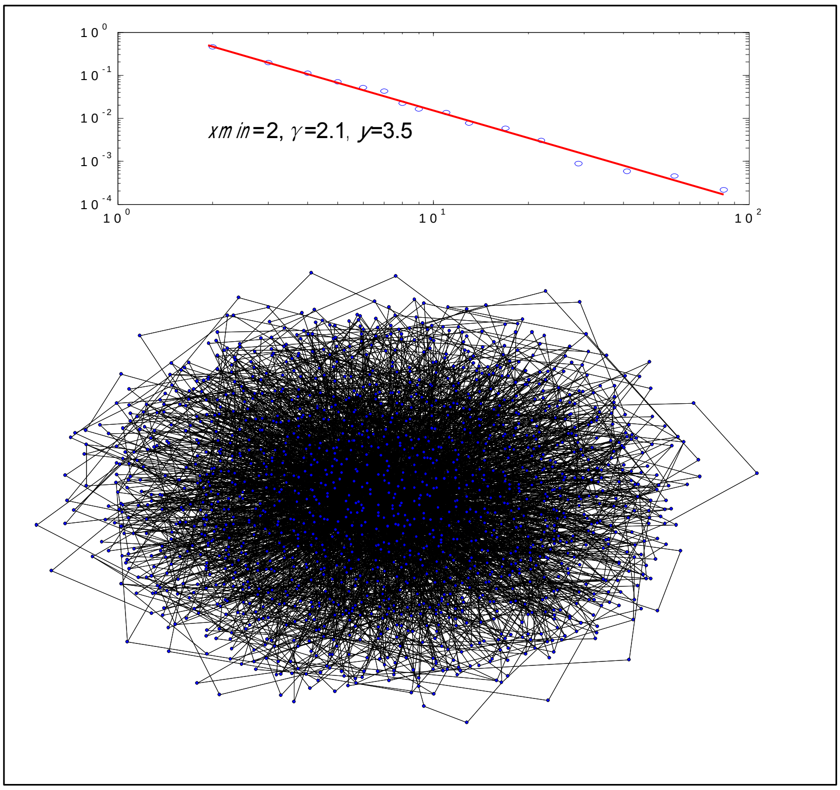

Fig. S31. Group (b). A compact scale-free network with *xmin*=2, γ=2 and *c*=3.5

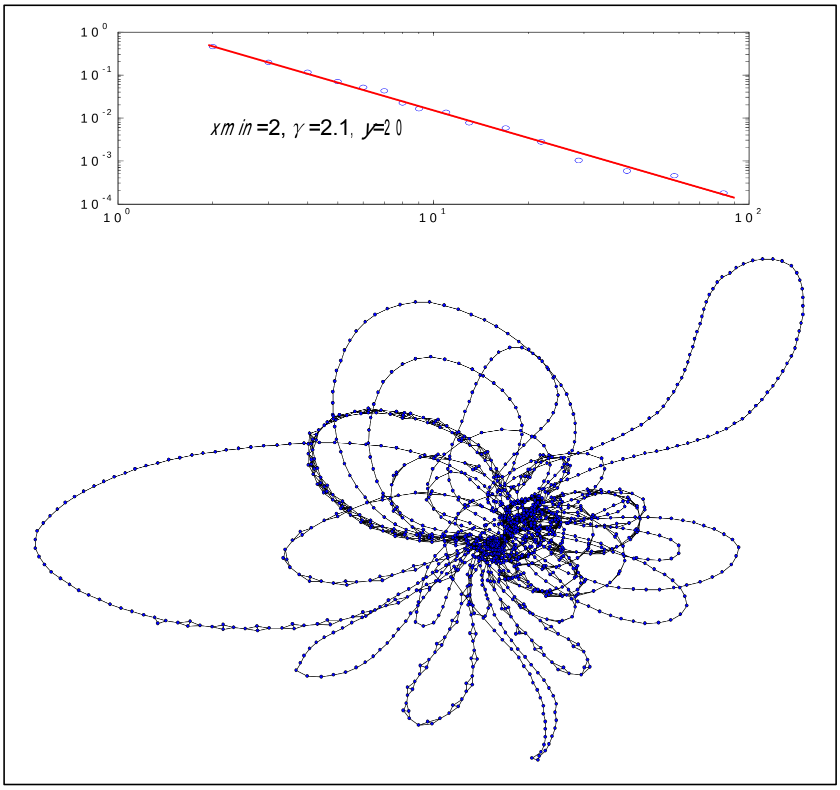

Fig. S32. Group (c). A fractal network with *xmin*=2, γ=2 and *c*=20

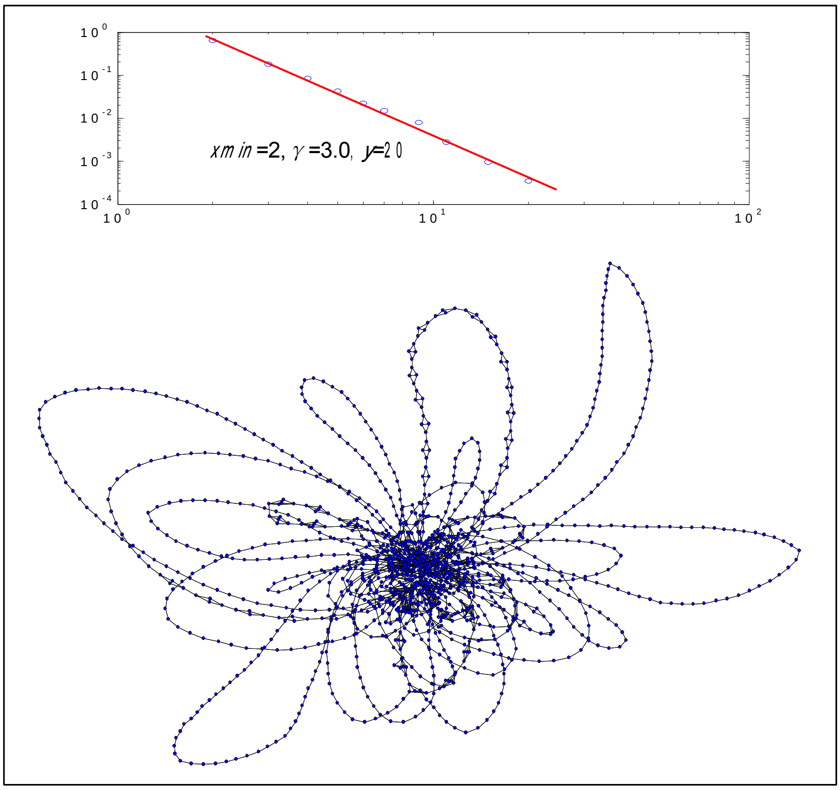

Fig. S33. Group (d). A fractal network with *xmin*=2, γ=3 and *c*=20

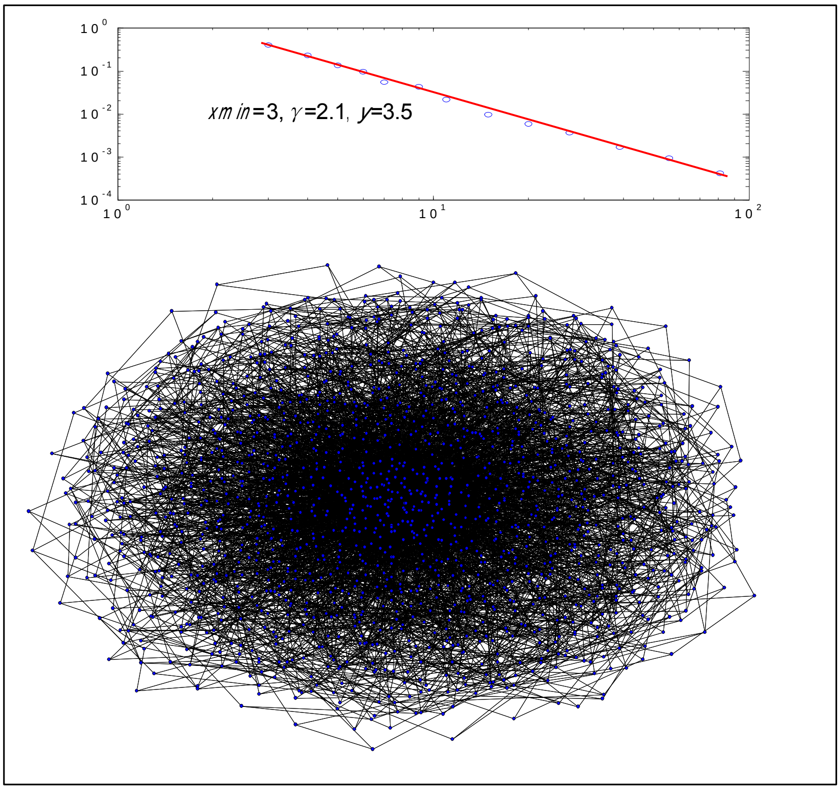

Fig. S34. Group (e). A small-world scale-free network with *xmin*=3, γ=2 and *c*=3.5

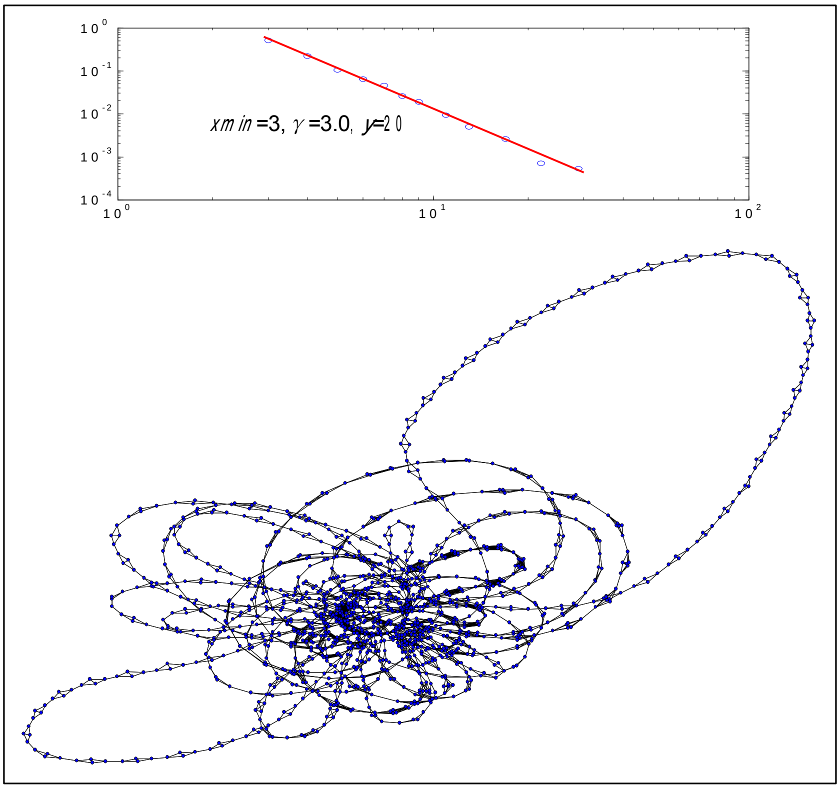

Fig. S35. Group (f). A fractal network with *xmin*=3, γ=3 and *c*=20

For all the demonstrated networks in Fig. S30- Fig. S35, the scale-free pattern is tested statistically[7], and the corresponding results are listed in Table S4.

Table S4. Estimated distribution of six groups of experiments

group		power law	exp.	stretched exp.	log-normal	power law + cutoff	status
Fig. S30	LR	-	391.515	-1.857	-9.432	-1.355	weak
	p	0.679	0.000	0.000	0.002	0.176	
Fig. S31	LR	-	235.510	210.356	222.394	7.064	good
	p	0.430	0.000	0.000	0.000	0.000	
Fig. S32	LR	-	40.558	51.472	38.261	7.076	good
	p	0.211	0.000	0.000	0.000	0.000	
Fig. S33	LR	-	240.813	211.420	183.117	7.126	good
	p	0.417	0.000	0.000	0.000	0.000	
Fig. S34	LR	-	214.133	269.678	159.591	11.836	good
	p	0.630	0.000	0.000	0.000	0.000	
Fig. S35	LR	-	63.940	63.454	34.162	3.134	good
	p	0.590	0.000	0.000	0.000	0.002	

As shown in Table S4, the scale-free properties were obvious for all of the demonstrated networks and the results were satisfactory.

On Arbitrary Traits and their Combinations

The optimisation algorithm used in the present study can be generalised to deal with arbitrary traits and the combinations of traits. Thus, scale-free properties and other traits can be explained by optimisation[8].

The optimisation algorithm uses a set of samples of the power law distributions to guide the optimisation direction. In fact, the other distributions are also suitable, as the sampling process only depends on the density functions. Here, the density functions were known. When the ideal samples were obtained, we can optimise the degree distribution of networks to match the ideal samples.

Furthermore, according to the Lagrangian relaxation method, the proposed method can account for multiple constraints. Therefore, when we append additional constraints into the original functions, the whole framework does not change, and any traits other than the degree

distribution can be depicted by optimisation.

For example, if we want the clustering coefficient of the obtained network to be 0.1, i.e., *cc* is 0.1, then we can rewrite the original equation as Equation (S24).

$$\begin{cases} \min F_1(A) = \sum_{i=1}^{N} x_i \\ \max F_2(A) = \sum_{i=1}^{N}\sum_{j=1}^{N} x_i^a x_j^b \delta_{ij} \end{cases}$$

subject to

$$y = c \qquad \text{(S24)}$$

$$cc(A) = 0.1$$

$$N > x_i \geq \text{xmin}$$

If we need to change the distribution, we can assume that the expected degrees are $K_1, K_2, \ldots, K_N$, and the equation can be rewritten as Equation (S25).

$$\min\ g(A) = \sum_{i=1}^{N} (x_i - K_i)^2$$

subject to

$$y = c \qquad \text{(S25)}$$

$$cc(A) = 0.1$$

$$N > x_i \geq \text{xmin}$$

We demonstrated this method with the log-normal degree distribution. To simplify the demonstration, we chose the simpler situation as shown in Equation (S26).

$$\min\ g(A) = \sum_{i=1}^{N} (x_i - K_i)^2$$

subject to (S26)

$$y = c$$

$$N > x_i \geq \text{xmin}$$

The probability density function of the log-normal distribution can be expressed as Equation (S26').

$$f_X(x; u, \sigma) = \frac{1}{x\sigma\sqrt{2\pi}} e^{-\frac{(\ln x - u)^2}{2\sigma^2}}, x > 0 \quad \text{(S26')}$$

We used the proposed fast algorithm to solve the optimisation problem defined by Equation

(S26).

Table S5. The parameters on the log-normal distribution

group	*xmin*	*xmax*	*u*	σ	c
(I)	2	150	3	1	7.3
(II)	2	150	3	1	3.5
(III)	2	150	4	1	7.3
(IV)	2	150	4	1	3.5

For every group of parameters, we performed the algorithm ten times to check the robustness of the fast algorithm. The experimental results showed that the topology of the networks was similar. Therefore, we selected the first run, as shown in Fig. S36 - Fig, S39.

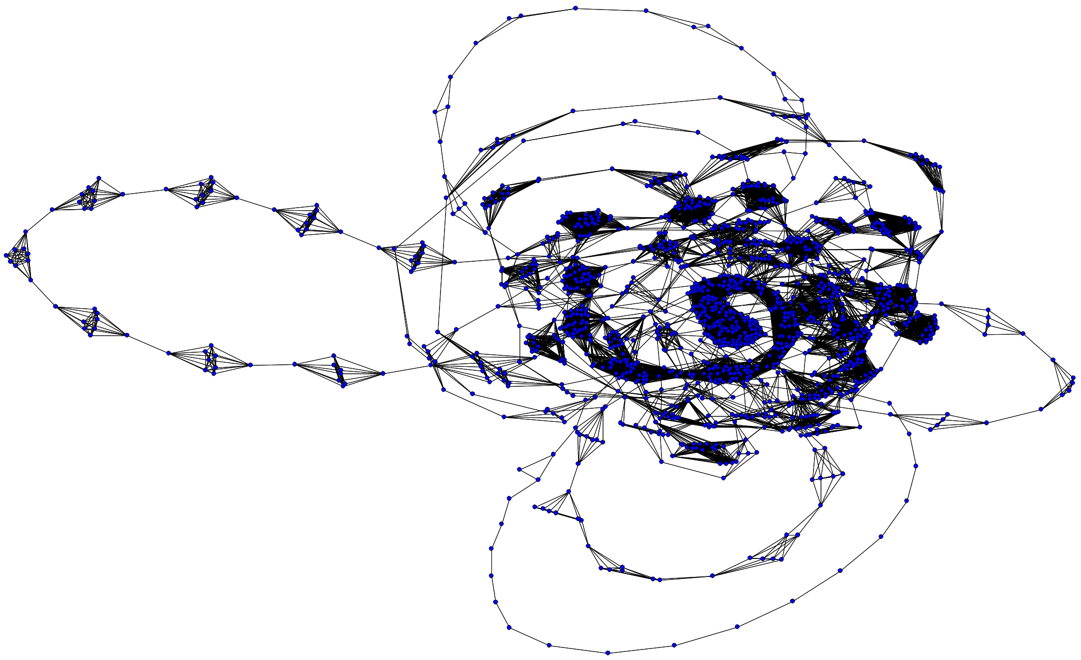

Fig. S36. Group (I). A log-normal network with *xmin*=2, u=3, and *c*=7.3

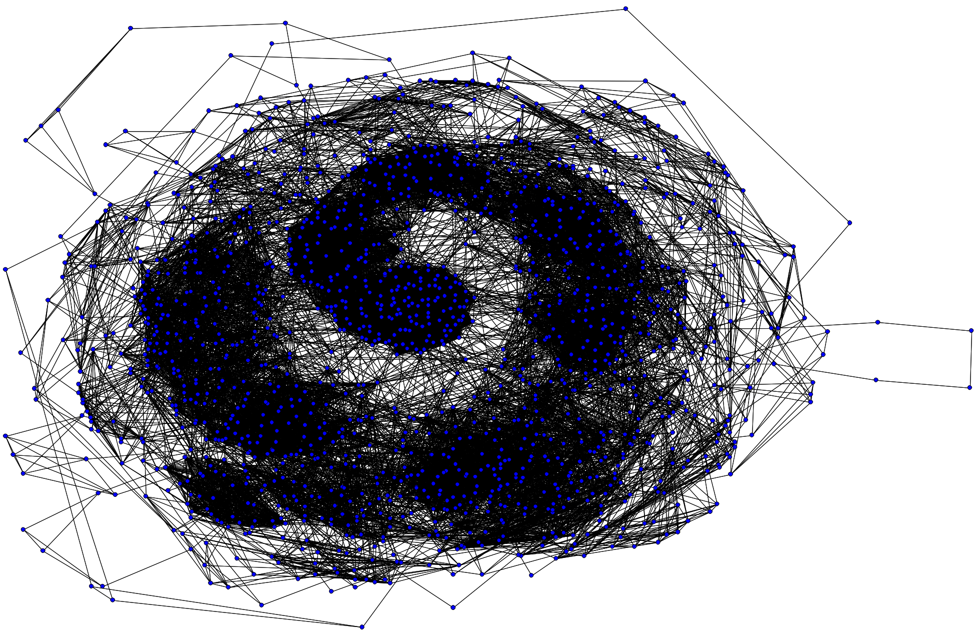

Fig. S37. Group (II). A log-normal network with *xmin*=2, u=3, and c=3.5

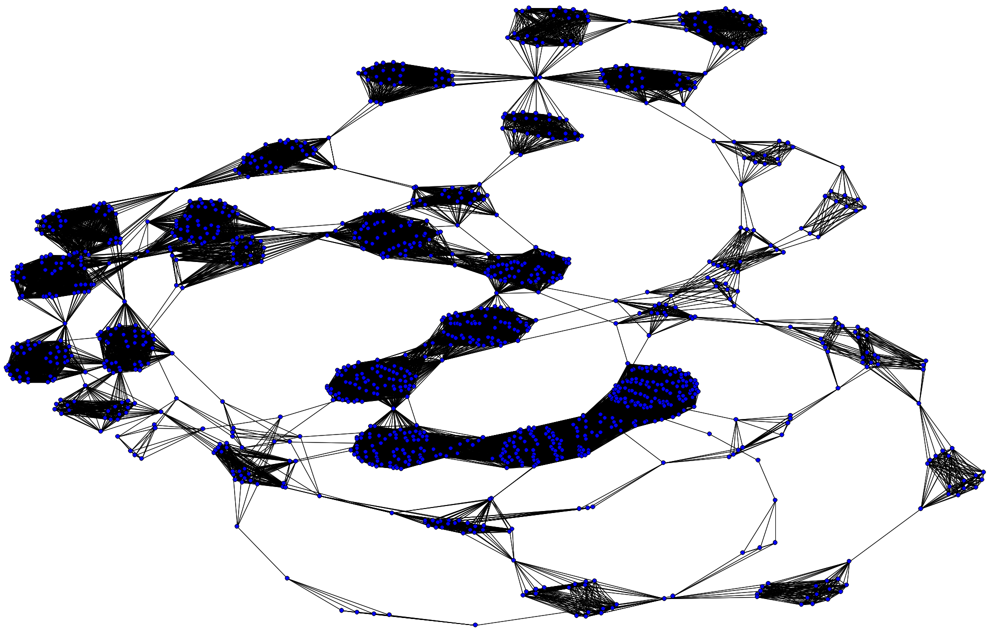

Fig. S38. Group (III). A log-normal network with *xmin*=2, u=4, and c=7.3

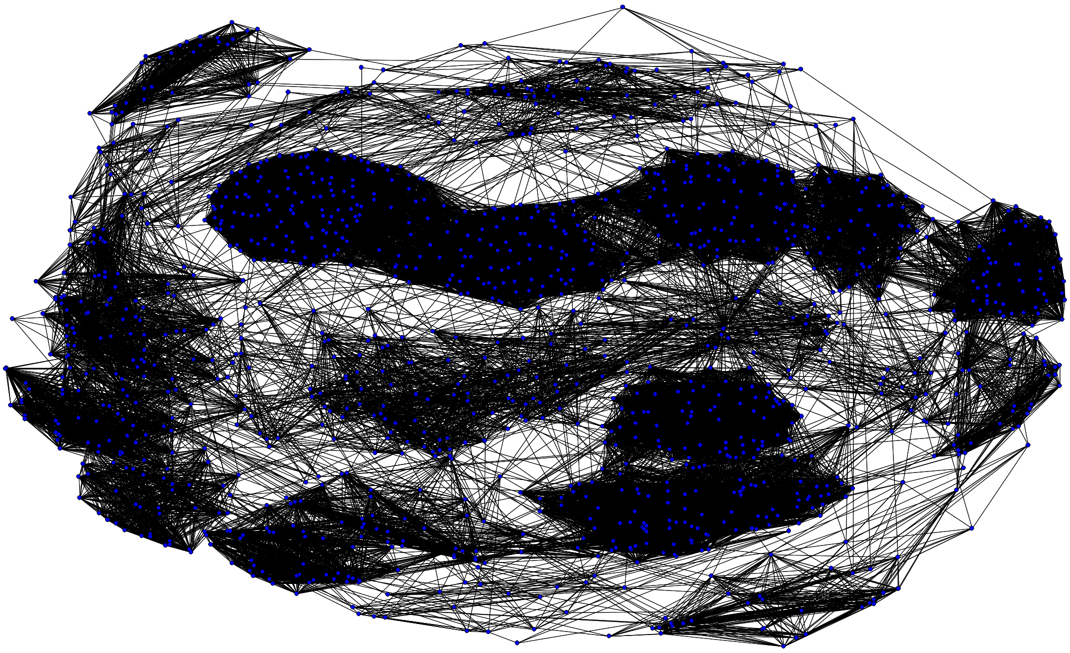

Fig. S39. Group (III). A log-normal network with *xmin*=2, u=4, and c=3.5

For these four demonstrated networks, we analysed their probability density functions and determined the cumulative distribution of the degree distribution. Because the curves of degree distributions of group (I) and group (II) and group (III) and group (IV) were similar, respectively, redundant figures have been omitted.

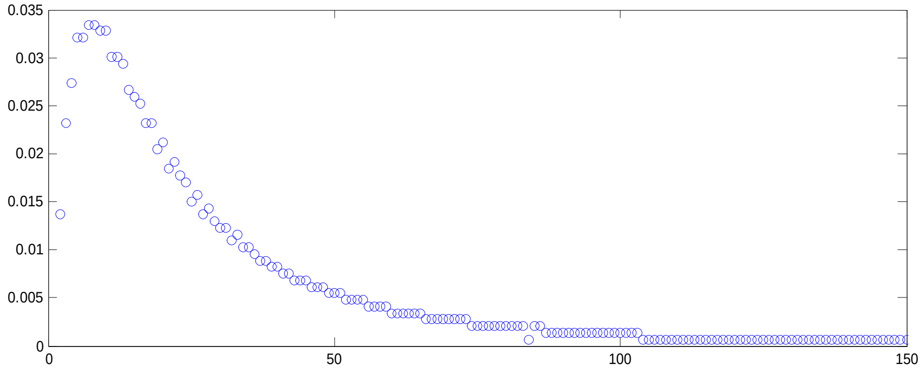

Fig. S40. The probability density function of the network in group (I)

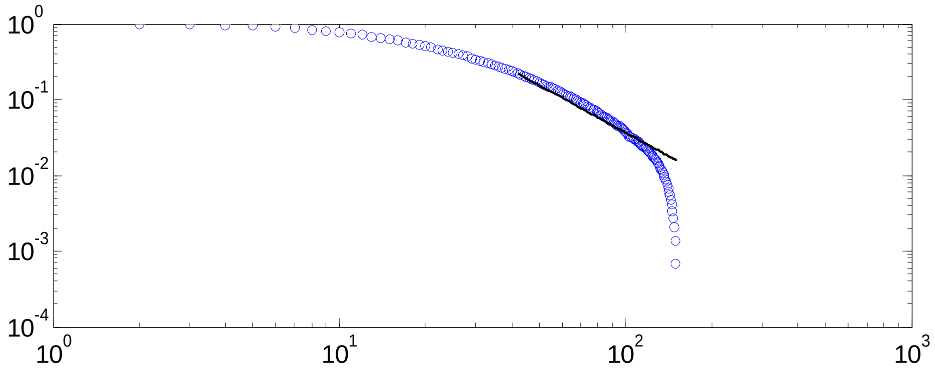

Fig. S41. The cumulative distribution function of the network in group (I) on a log-log scale

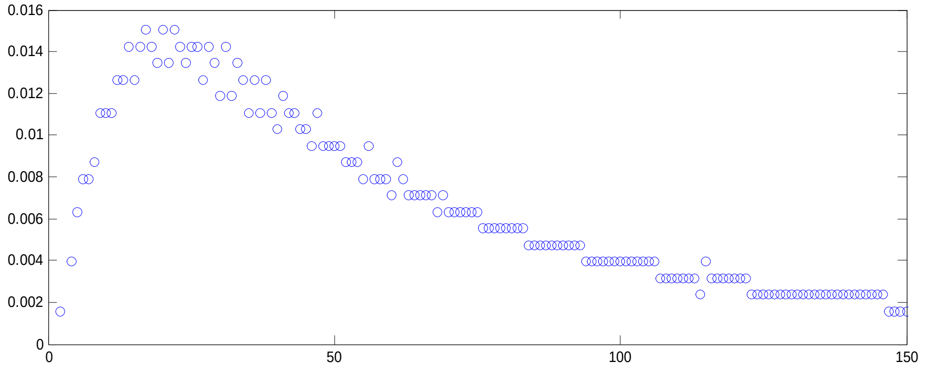

Fig. S42. The probability density function of the network in group (III)

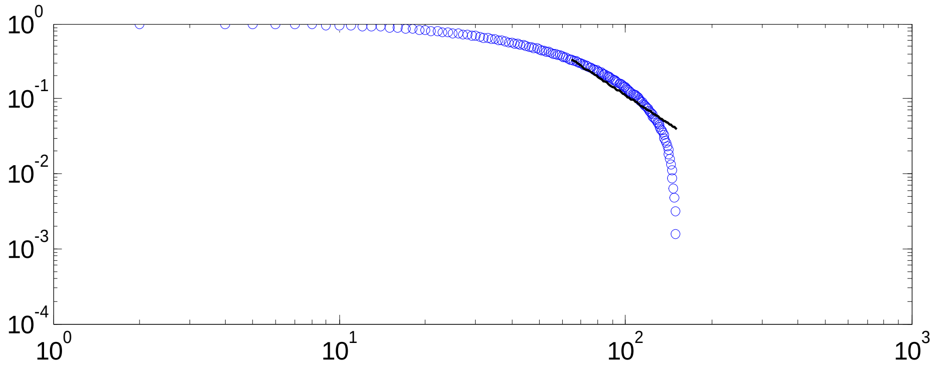

Fig. S43. The cumulative distribution function of the network in group (III) on a log-log scale

In general, the method proposed herein can deal with arbitrary distributions as well as the other traits with the constraints.

On the Fractal Network

Song et al. defined the fractality as the occurrence of a power law relationship between the box number and the maximum box diameter. This definition of fractality is based on the length transformation, as shown in Equation (S27). When using a modular hierarchical structure to construct fractal networks, if the box-covering method absorbs the modules within a maximum box diameter, then the box number has a power law relationship with the maximum box diameter. Here, the hub nodes repulse other hub nodes.

$$N_B \; : \; l_B^{\,d_B} \quad \text{(S27)}$$

However, the modular hierarchical structure is not the only way to satisfy the fractality described by Song et al. When the marginal nodes are repulsed from the central node, the network is considered fractal when the box numbers respond with the power law relationship for any maximum box diameter.

With the box-covering method, a few examples are used to illustrate the fractal networks with hub aggregation.

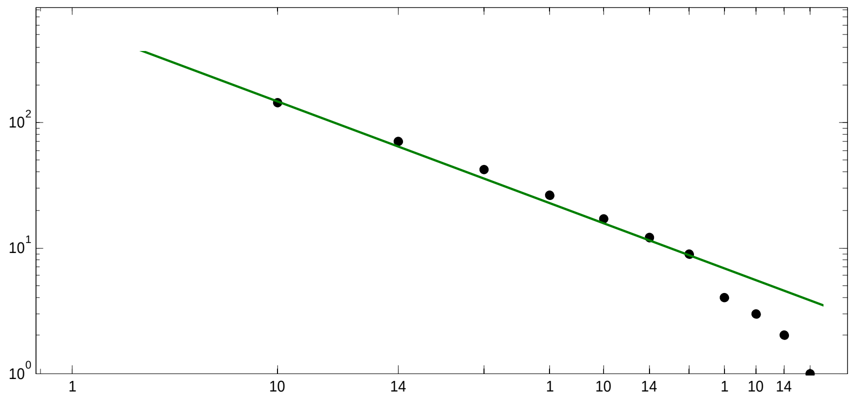

Fig. 44. The fractality of Fig. S18 With *xmin*=3, c=5 and N=300

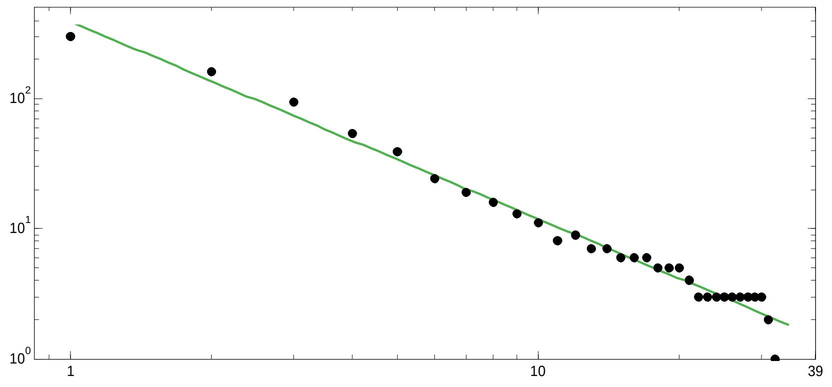

Fig. S45. The fractality of Fig. S14 with *xmin*=2, c=7 and N=300

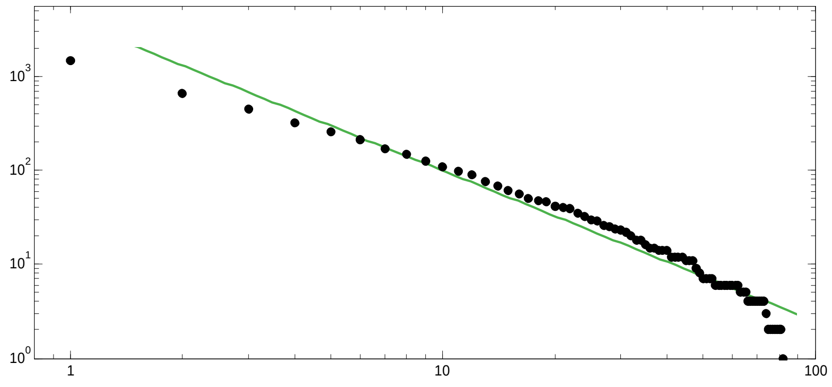

Fig. S46. The fractality of Fig. S33 with *xmin*=2, c=20 and N=1500

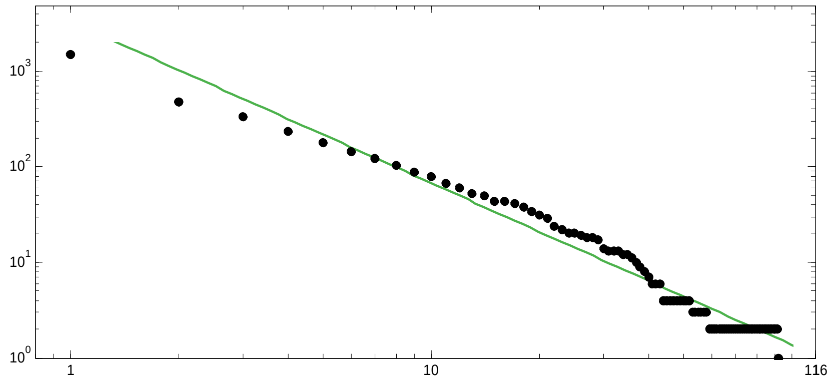

Fig. S47. The fractality of Fig. S35 with *xmin*=3, c=20 and N=1500

According to the mathematics, the self-similarity can be expressed as shown in Equation (S28).

$$f(x)\ :\ x^{-r} \qquad \text{(S28)}$$

Therefore, the fractality or self-similarity described by Song et al. is a type of similarity over the box diameter, which can also be described as structural self-similarity over diameter.

Additionally, scale-free networks have a probabilistic similarity over varying degree, which we refer to as degree self-similarity.

As to the other measures, such as the clustering coefficient, different self-similarity can be defined. However, for a specific network, a new method such as the box-covering method described by Song et al. can be used to test the self-similarity of the generated fractal networks.

Because the average shortest path length is also a measure of the length, the fractality over the box diameter can be approximated when applying the box-covering method to the demonstrated networks, as shown in Fig. S44- Fig. S47.

Moreover, there are some fractal and high assortative networks in the real world, such as the Movie actors, and the explanation on fractality and assortativity are shown in the paper “A fractal and scale-free model of complex networks with hub attraction behaviors”, arxiv: 1311.3087.

On the community-structure networks with multiple communities

To satisfy community networks with multiple communities, similarity distance is termed as the distance in real world, such as geographical distance, interests and preferences. We formulate the similarity distance between two nodes i and j as g_{ij}, and $g_{ij}=k$ when the two nodes belong to different categories, while $g_{ij}=t$ for the two nodes in the same category. Here a and b are constants, and $a>b$. We only consider the simplest form that a and b are constants, though we can also define g_{ij} in a complex form. According to the definition of the similarity distance between nodes, the similarity distance of a network l can be defined as $l=\frac{1}{2}\sum_{i\neq j}^{N} g_{ij}\delta_{ij}$. We can rewrite the distance of the network as Equation (S29), where l and y are respectively the similarity distance and average topological distance of the resultant networks .

$$y' = l + y \qquad \text{(S29)}$$

Similar to equation (S19), in S(30) where c is still the average topological distance, while Δ represents the similarity distance of a desired network.

$$c' = \Delta + c \qquad \text{(S30)}$$

Similar to the transforming process in "On the revised model for community-structure scale-free networks", the revised model can be rewritten as Equation (S32).

$$\begin{cases} \min F_1(A) = \sum_{i=1}^{N} x_i \\ \max F_2(A) = \sum_{i=1}^{N}\sum_{i=1}^{N} x_i^a x_j^b \delta_{ij} \end{cases}$$
$$\text{subject to} \qquad \text{(S32)}$$
$$y' = c'$$
$$N > x_i \geq \text{xmin}$$

As S1 can generate optimal scale-free networks with proper *c*, and Equation S32 is similar to S1, so it is easily to prove that the revised model S32 can generate optimal networks with community-structure and scale-free property.

In Fig. S49-S53, we present five community-structure and scale-free networks with multiple communities. In the five figures, the similarity distance between nodes is set with k=10 and t=1, the other parameters are shown in Table S6.

Table S6. The parameters for community networks with multiple communities

group	*xmin*	γ	*N*	*E*	*c*	*l*
Fig. S49	2	2	300	761	4	905
Fig. S50	2	2	300	761	4	806
Fig. S51	2	3	300	678	4	713
Fig. S52	2	2	3483	12747	6	12828
Fig. S53	1	2	18000	24625	4.2	24652

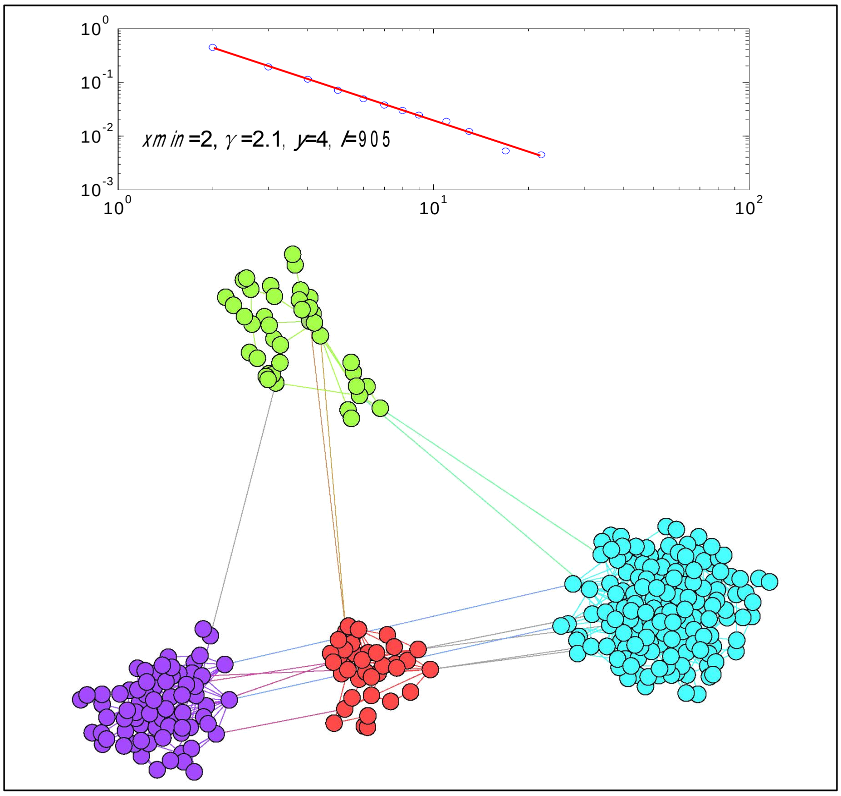

Fig. S49. A community network with *xmin*=2, γ=2, *c*=4 and *l*=905

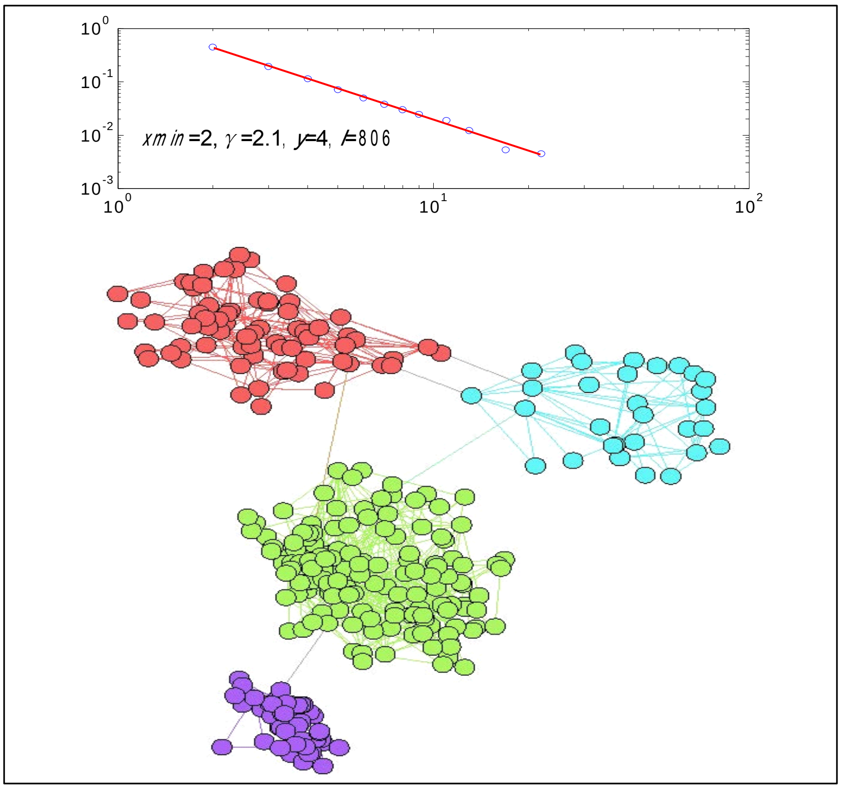

Fig. S50. A community network with *xmin*=2, γ=2, *c*=4 and *l*=806

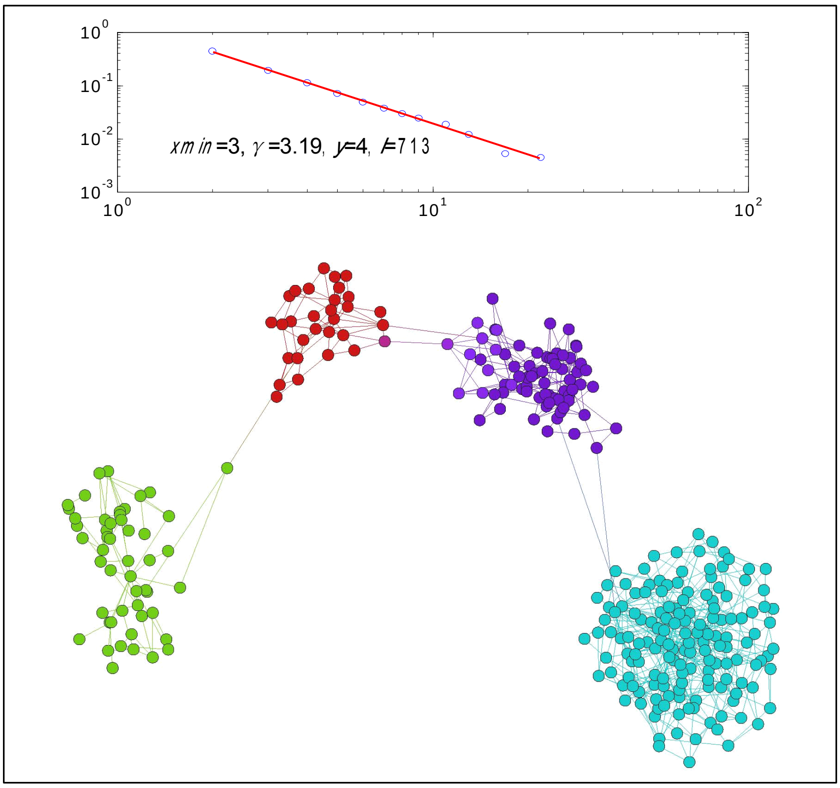

Fig. S51. A community network with *xmin*=3, γ=3, *c*=4 and *l*=713

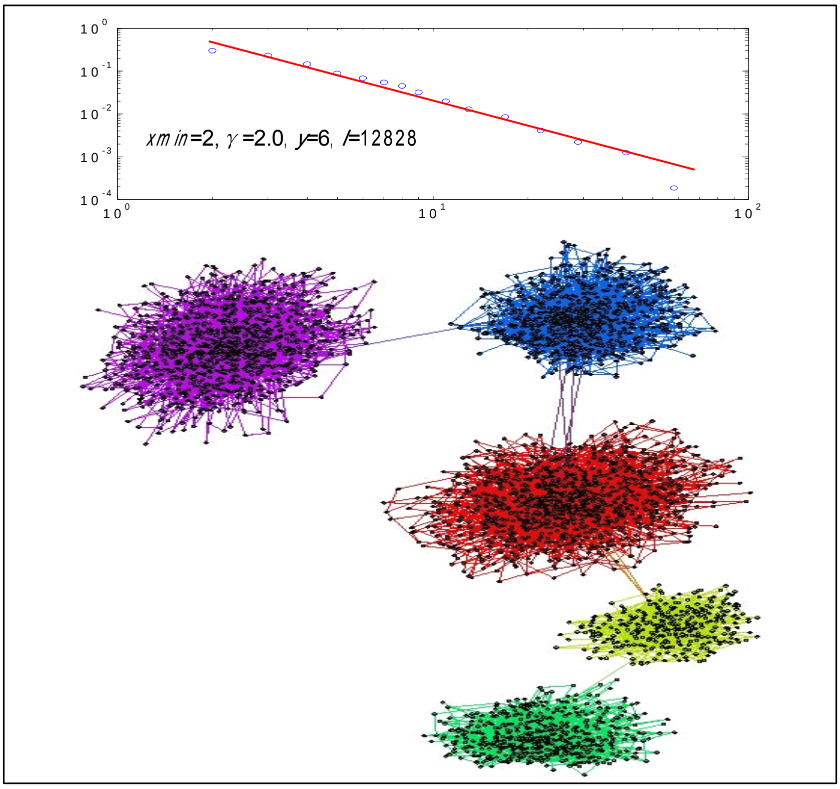

Fig. S52. A community network with *xmin*=2, γ=2, *c*=6 and *l*=12828

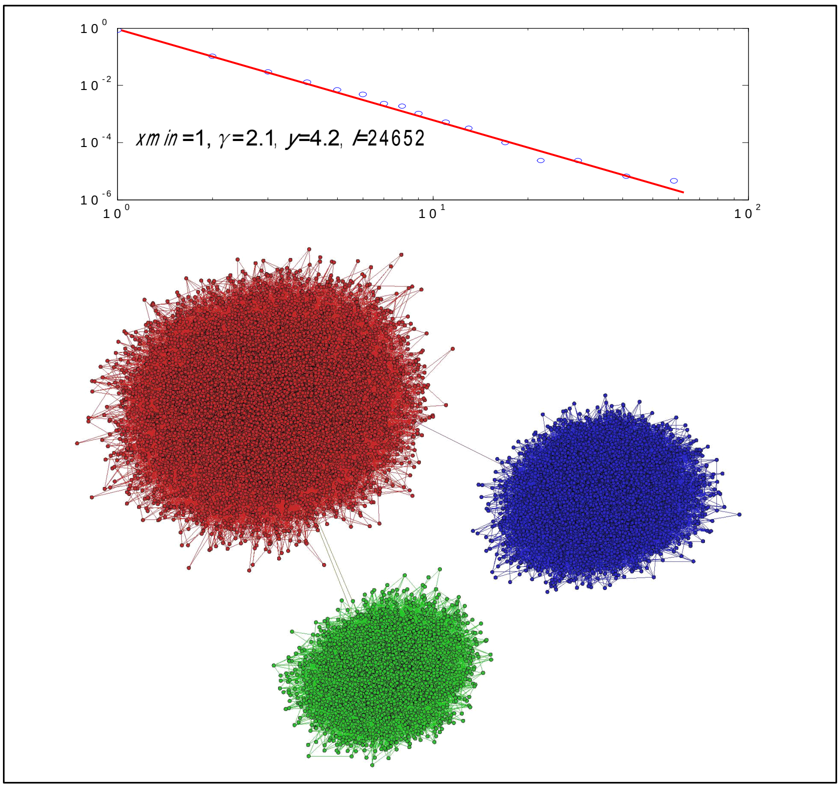

Fig. S52. A community network with *xmin*=1, γ=2, *c*=4.2 and *l*=24652

Acknowledgments

We thank Clauset, A. Borgatti, S.P. Everett, M.G. and Freeman, L.C. for providing plotting tools.

References

1. Horn, J., Nafpliotis, N. & Goldberg, D. E. in Proceedings of the First IEEE Conference on Evolutionary Computation, IEEE World Congress on Computational Intelligence 82-87 (1994).
2. Börzsönyi, S., Kossmann, D. & Stocker, K. in Proceedings of the 17th International Conference on Data Engineering 421-430 (IEEE Computer Society, 2001).
3. Li, H. & Zhang, Q. Multiobjective Optimization Problems With Complicated Pareto Sets, MOEA/D and NSGA-II. IEEE Transactions On Evolutionary Computation 13, 284-302 (2009).
4. Johnson, D. B. Efficient algorithms for shortest paths in sparse networks. Journal of the ACM 24, 1-13 (1977).
5. Floyd, R. W. Algorithm 97: Shortest Path. Communications of the ACM 5, 345 (1962).
6. Song, C., Halin, S. & Makse, H. A. Self-similarity of complex networks. Nature 433, 392-395 (2005).
7. Virkar, Y. & Clauset, A.Power-law distributions in binned empirical data. (2012).arXiv:1208.3524
8. Papadopoulos, F., Kitsak, M., Serrano, M. Á., Boguñá, M. & Krioukov, D. Popularity versus similarity in growing networks. Nature 489, 537–540 (2012).
